# Discovery and validation of a three-gene signature to distinguish COVID-19 and other viral infections in emergency infectious disease presentations: a case-control and observational cohort study

**DOI:** 10.1016/S2666-5247(21)00145-2

**Published:** 2021-11

**Authors:** Ho Kwong Li, Myrsini Kaforou, Jesus Rodriguez-Manzano, Samuel Channon-Wells, Ahmad Moniri, Dominic Habgood-Coote, Rishi K Gupta, Ewurabena A Mills, Dominique Arancon, Jessica Lin, Yueh-Ho Chiu, Ivana Pennisi, Luca Miglietta, Ravi Mehta, Nelofar Obaray, Jethro A Herberg, Victoria J Wright, Pantelis Georgiou, Laura J Shallcross, Alexander J Mentzer, Michael Levin, Graham S Cooke, Mahdad Noursadeghi, Shiranee Sriskandan

**Affiliations:** a Department of Infectious Disease, Imperial College London, London, UK; b Medical Research Council Centre for Molecular Bacteriology & Infection, Imperial College London, London, UK; c National Institute for Health Research Health Protection Research Unit in Healthcare-associated Infection & Antimicrobial Resistance, Imperial College London, London, UK; d Department of Electrical & Electronic Engineering, Imperial College London, London, UK; e Centre for Bio-Inspired Technology, Imperial College London, London, UK; f Institute of Global Health, University College London, London, UK; g Institute of Health Informatics, University College London, London, UK; h Division of Infection and Immunity, University College London, London, UK; i Imperial College Healthcare NHS Trust, London, UK; j Wellcome Centre for Human Genetics, University of Oxford, Oxford, UK

## Abstract

**Background:**

Emergency admissions for infection often lack initial diagnostic certainty. COVID-19 has highlighted a need for novel diagnostic approaches to indicate likelihood of viral infection in a pandemic setting. We aimed to derive and validate a blood transcriptional signature to detect viral infections, including COVID-19, among adults with suspected infection who presented to the emergency department.

**Methods:**

Individuals (aged ≥18 years) presenting with suspected infection to an emergency department at a major teaching hospital in the UK were prospectively recruited as part of the Bioresource in Adult Infectious Diseases (BioAID) discovery cohort. Whole-blood RNA sequencing was done on samples from participants with subsequently confirmed viral, bacterial, or no infection diagnoses. Differentially expressed host genes that met additional filtering criteria were subjected to feature selection to derive the most parsimonious discriminating signature. We validated the signature via RT-qPCR in a prospective validation cohort of participants who presented to an emergency department with undifferentiated fever, and a second case-control validation cohort of emergency department participants with PCR-positive COVID-19 or bacterial infection. We assessed signature performance by calculating the area under receiver operating characteristic curves (AUROCs), sensitivities, and specificities.

**Findings:**

A three-gene transcript signature, comprising *HERC6, IGF1R*, and *NAGK*, was derived from the discovery cohort of 56 participants with bacterial infections and 27 with viral infections. In the validation cohort of 200 participants, the signature differentiated bacterial from viral infections with an AUROC of 0·976 (95% CI 0·919−1·000), sensitivity of 97·3% (85·8−99·9), and specificity of 100% (63·1−100). The AUROC for C-reactive protein (CRP) was 0·833 (0·694−0·944) and for leukocyte count was 0·938 (0·840−0·986). The signature achieved higher net benefit in decision curve analysis than either CRP or leukocyte count for discriminating viral infections from all other infections. In the second validation analysis, which included SARS-CoV-2-positive participants, the signature discriminated 35 bacterial infections from 34 SARS-CoV-2-positive COVID-19 infections with AUROC of 0·953 (0·893−0·992), sensitivity 88·6%, and specificity of 94·1%.

**Interpretation:**

This novel three-gene signature discriminates viral infections, including COVID-19, from other emergency infection presentations in adults, outperforming both leukocyte count and CRP, thus potentially providing substantial clinical utility in managing acute presentations with infection.

**Funding:**

National Institute for Health Research, Medical Research Council, Wellcome Trust, and EU-FP7.

## Introduction

There has been extensive interest in biomarkers that discriminate between bacterial and viral infections in emergency medicine, primarily fuelled by the aspiration to reduce inappropriate prescribing of antibacterial agents.^1^ The COVID-19 pandemic has highlighted a further need for tests that can trigger infection control and antiviral interventions before a specific diagnosis is available.

Molecular diagnostic tests for viruses are often used only if participants are admitted to hospital and, in the setting of an emerging pandemic, such tests might not be available if the causal pathogen is yet to be identified, or delayed through over-centralisation, rationing of testing, or issues with single suppliers.^2^, ^3^ The COVID-19 pandemic has brought these issues into sharp focus, presenting health-care systems with an immense diagnostic challenge. Molecular diagnostic tests for SARS-CoV-2 were not widely available at the start of the pandemic,^4^ underlining the value of a readily available agnostic blood test (ie, does not require previous knowledge of any specific pathogenic agent) to detect viral diseases, such as COVID-19, by host response. As the pandemic developed, notwithstanding delays in test availability, COVID-19 posed additional challenges, because the clinical features and routine laboratory diagnostic tests greatly overlapped with those of bacterial sepsis.^5^ Perhaps the greatest challenge has been the association of COVID-19 with elevated C-reactive protein (CRP). Although molecular tests for SARS-CoV-2 appear robust, the phenomenon of the SARS-CoV-2 PCR-negative participant who manifests disease consistent with COVID-19 is well reported.^6^, ^7^, ^8^ There is the possibility that viral genetic variation in the future could render PCR or antigen-based tests for SARS-CoV-2 unreliable. Furthermore, as the pandemic recedes, COVID-19 could be considered less probable in the differential diagnosis. There is a pressing need for a ready-to-use test that is available to all and can discriminate viral infections, such as COVID-19, from other infections.

**Research in context**

**Evidence before this study**

Differentiation of infections is a major challenge in emergency settings, where decisions to prescribe antibiotics to potentially save life must be weighed against the risk of aiding antimicrobial resistance or of complications through over-use. Viral diagnoses are perceived to have few immediate treatment consequences, thus were seldom actively sought until the COVID-19 pandemic. Several molecular diagnostic biomarkers to distinguish between viral and bacterial infection have been proposed but none are in routine use. We searched PubMed for clinical studies published in English between database inception and April 1, 2021, using the search terms “bacterial” AND “viral” AND “blood” AND (“RNAseq” OR “transcriptomic” OR “transcriptome” OR “gene expression”) AND (“signature” OR “diagnosis” OR “classification” OR “classifier”). Our search returned more than 70 papers, and five gene expression signatures that distinguish between bacterial and viral infections, although none were derived and validated in adult emergency settings, and none have been validated in patients with COVID-19.

**Added value of this study**

Our study provides a unique snapshot of gene expression in a large cohort of well phenotyped adults at the point of admission to an emergency department with suspected sepsis. A simple three-gene PCR signature was derived, which has superior ability to differentiate viral from other infection presentations compared with existing biomarkers, such as C-reactive protein and leukocyte count. The signature was validated on a prospective cohort of real-world emergency infectious disease admissions. The signature was further validated on PCR-positive COVID-19 admissions to the emergency department, a group where RT-qPCR testing via nasopharyngeal sampling alone can be inaccurate. To our knowledge, this analysis represents the first host gene signature to be validated for COVID-19, providing proof of principle that point-of-care gene expression-based diagnostics can support decision making in acute and ambulatory emergency settings.

**Implications of all the available evidence**

Infection-related primary diagnoses represent almost 20% of emergency department presentations in the USA. There is an expanding menu of biomarker and gene expression signatures that can differentiate infection from inflammation, and viral from bacterial infection. Viral infection is characterised by a unique host gene expression profile that is highly specific and is unlikely to be affected by variation in viral genome. Our study has shown that such profiles can be reduced to a simple three-gene, rapid PCR-based signature that can distinguish non-SARS-CoV-19 and SARS-CoV-19 viral infections from other emergency sepsis presentations. Few gene expression signatures enable easy translation to point-of-care test. Our work provides a roadmap to a fingerprick test that could ultimately distinguish COVID-19 from other viral and bacterial disease presentations in adults, providing an important asset in the current pandemic and a tool to guide future antimicrobial prescribing. The three-gene signature we discovered could allow for improved stratification of anti-infective prescribing among participants with respiratory illness, contribute to earlier diagnosis, and provide more targeted infection control strategies in the COVID-19 era.

Blood transcriptomic biosignatures have the potential to be translated into point-of-care diagnostic tests and have shown promise in distinguishing between patients with and without infection and between those with bacterial and with viral infection, as well as severity stratification.^9^, ^10^, ^11^, ^12^, ^13^, ^14^, ^15^

We aimed to characterise the differential gene expression in a unique cohort of patients who presented at an emergency department with suspected infections. We hypothesised that a minimal diagnostic signature identified in the blood of patients with confirmed viral and bacterial infections could be used to discriminate these infections via an RT-qPCR diagnostic assay. We then aimed to assess the performance of the RT-qPCR assay in the identification of viral infections from the large mix of adult patients presenting to the emergency department with suspected infection in a prospectively recruited cohort of patients who required hospitalisation, and further evaluated the assay in an additional emergency department cohort of COVID-19 patients who required hospitalisation.

## Methods

### Study design and participants

Individuals (aged ≥18 years) presenting with suspected infection to an emergency department were prospectively recruited at a major teaching hospital in the UK as part of the Bioresource in Adult Infectious Diseases (BioAID).^16^ Individuals were eligible if they had a clinical syndrome sufficient to warrant blood culture testing. Whole-blood RNA was obtained at the point of presentation, in conjunction with demographic and clinical data.

Eligible participants were recruited into three separate cohorts: the discovery cohort and two validation cohorts. The discovery cohort comprised participants identified as having definite categories of infections (definite bacterial, definite viral, or no infection) and was used to derive a novel gene expression signature. Two separate validation cohorts were used to validate the signature: a pre-COVID-19 pandemic cohort and a COVID-19 pandemic cohort.

Ethical approval was granted by the South Central–Oxford C national research ethics committee to obtain deferred consent from participants from whom an RNA specimen had been collected (14/SC/0008 and 19/SC/0116).^16^

### Discovery cohorts for RNA sequencing analysis

The discovery cohort (case-control design) comprised participants recruited between Sept 15, 2014, and April 28, 2017. We used National Health Service (NHS) hospital pathology and patient administration databases to identify 30 participants from each of the following categories: microbiologically confirmed Gram-positive or Gram-negative bacteraemia; a positive viral diagnostic test in the context of acute admission; and those with no positive microbial diagnostic test, no infection-related International Classification of Diseases-10 diagnostic codes from BioAID admission, and no empirical antiviral or antimicrobial treatment lasting more than 48 h. When more than 30 participants were identified in any category, random selection was applied using Microsoft Excel. Using whole-blood samples already collected on admission, RNA sequencing analysis was done. In parallel, full case records of each participant were reviewed by two independent specialists (HKL and SS) to confirm categorisation into specific infection categories (appendix p 11). Differential gene expression analyses, with additional filtering criteria (absolute log_2_ fold-change >1, adjusted p<0·05, and mean expression value; appendix p 14), between all categories and feature selection (using forward selection-partial least squares [FS-PLS] or elastic net) were done (appendix pp 4−7, 23).

### Three-gene signature derivation

All participants with definite bacterial or viral infection were included in signature discovery (appendix pp 4−5). We used a FS-PLS method to identify a minimal diagnostic signature, resulting in a logistic regression model. FS-PLS selects for minimally correlated genes and has previously been described in detail.^12^, ^17^ We calculated the FS-PLS score into a single metric using weighted sums of log_2_ gene counts (appendix pp 6, 29).

To assess the generalisability of the diagnostic signature, we assessed its performance in a publicly available microarray gene expression dataset of confirmed bacterial and viral infections (appendix pp 7, 31).^9^

### Pre-COVID-19 prospective validation cohort and three-gene signature evaluation

To test the hypothesis that the three-gene signature can discriminate bacterial infection from COVID-19, whole-blood RNA samples from a 6-month cohort of consecutively admitted BioAID participants (recruited between Sept 1, 2017, and Feb 28, 2018, >2 years before the onset of the first COVID-19 epidemic wave in the UK) were used to evaluate the performance of the gene expression signature using a Biomark HD high-throughput qPCR platform (Fluidigm, San Francisco, CA, USA; appendix pp 8−9). All emergency infectious disease admissions to hospital recruited to BioAID entered this cohort regardless of what their final diagnosis was. Participants had been previously categorised (appendix p 12) using clinical data as having definite viral, definite bacterial, probable viral, or probable bacterial infection. Participants with co-infections or lacking diagnostic certainty were categorised as indeterminate, and if no evidence of infection was found, participants were categorised as not infected or other infection (fungal, parasitic, or mycobacterial), reflecting real-life practice. The three-gene signature performance was assessed using area under receiver operating characteristic curve (AUROC) analysis.

### Decision curve analysis in the pre-COVID-19 prospective validation cohort

To determine how well the signature would perform in everyday clinical practice, we used decision curve analysis. We evaluated the clinical utility of each of leukocyte count, CRP, and the three-gene signature to identify definite and probable viral or bacterial infections among all participants in the entire pre-COVID-19 prospective validation cohort where all three measurements were available, including the indeterminate groups, thereby avoiding over optimistic estimates of test performance derived from case-control analyses. Decision curve analysis yields the net benefit of a given decision from the true positive rate (benefit) offset by the false positive rate (harm), weighted by the risk:benefit ratio of the intervention. The cutoff for the predictive value of a test that triggers an intervention (threshold probability) is used as a surrogate of perceived risk of the intervention (risk:benefit ratio). We considered a threshold probability of 50% to be the upper limit of acceptable risk for an intervention based on these tests. We used the rmda package (version 1.6) in R (version 3.6.3)^18^ for decision curve analyses. Full details are given in the appendix (pp 9−10).

### COVID-19 case-control validation cohort

We used NHS hospital pathology and patient administration databases to identify BioAID participants who had either definite COVID-19—defined as being RT-qPCR positive for SARS-CoV-2 and having acute COVID-19 symptoms (recruited between March 13 and May 5, 2020, in the first pandemic wave)—or definite bacterial infection (defined by bacteraemia, recruited between Nov 2, 2014, and Feb 5, 2020). 35 participants from each group were randomly selected using Microsoft Excel, omitting any participants previously included in RNA analyses (appendix p 13). Whole-blood RNA samples collected at admission to hospital underwent RT-qPCR for the novel diagnostic signature and were evaluated by AUROC analysis.

### Role of the funding source

The funders of the study had no role in study design, data collection, data analysis, data interpretation, or writing of the report.

## Results

**Figure 1:**
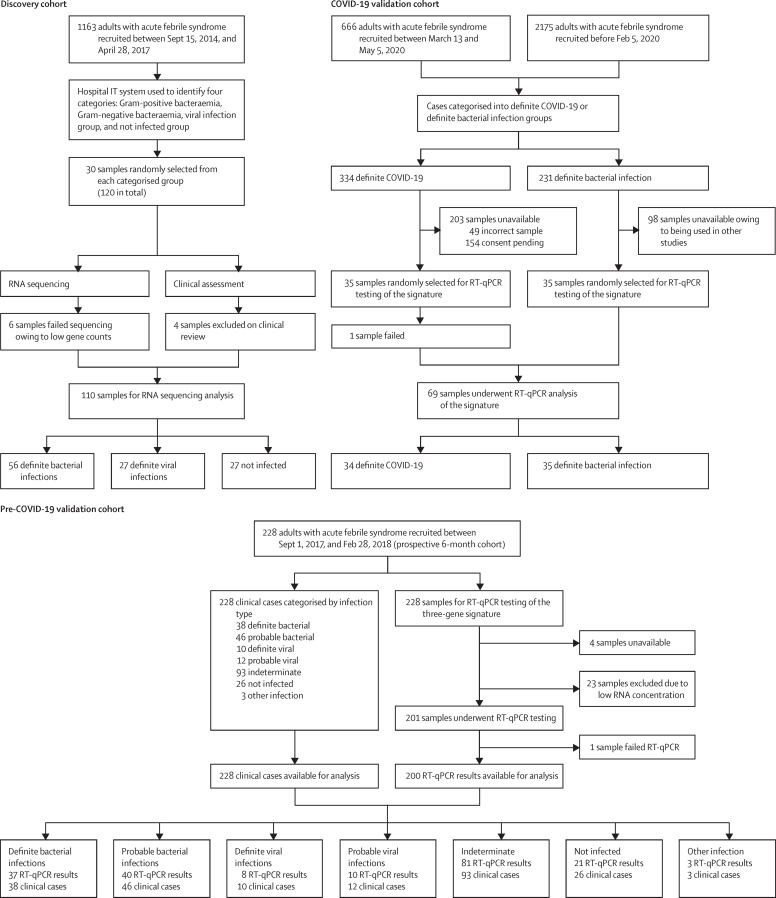
Study profile

To derive a minimal transcriptomic signature, an initial discovery cohort of 120 RNA samples was selected from participants of BioAID who presented to the emergency department with suspected sepsis.^16^ RNA sequencing was done from 30 participants with Gram positive bacteraemia, 30 with Gram-negative bacteraemia, 30 with a viral diagnosis, and 30 with no final microbiological diagnosis (appendix p 24). Pathogens identified in this discovery cohort are recorded in the appendix (p 25). A full case record review was done of all participants to assign them into definite bacterial, definite viral, or not infected categories (diagnostic algorithm, appendix p 11). Four participants were identified as having mixed or equivocal infection and, thus, were not included in our analysis. Five RNA samples were excluded due to a low RNA sequencing count and one failed RNA sequencing quality control, leaving 83 participants with definite bacterial or definite viral infection, and 27 classified as not infected, with RNA sequencing data to analyse (figure 1). Participants assigned as having definite viral infection were younger, had fewer comorbidities, and had lower leukocyte, neutrophil, and CRP counts than those with definite bacterial infections (appendix p 24).

Differential gene expression analysis comparing participants with definite bacterial or viral infection (n=83) against those who were categorised as having no infection (n=27) identified a list of 59 significant genes (adjusted p<0·01). 57 genes were overexpressed in the definite infection group but only two genes, *SLC24A2* and *CD1E*, were overexpressed in the no infection group (appendix pp 26−27).

To identify a signature that discriminates between bacterial and viral infection, data from the 56 participants with definite bacterial infection and 27 participants with definite viral infection underwent differential gene expression analysis. Between the two groups, 3469 genes were differentially expressed, of which 627 met additional filtering criteria (appendix p 14). Feature selection using elastic net identified 16 transcripts that discriminated bacterial from viral infection in the discovery cohort with an AUROC of 0·999 (95% CI 0·994–1·000), sensitivity of 96·4% (95% CI 92·9−100), and specificity of 100% (95% CI 96·3–100; appendix pp 15, 28, 30).

**Figure 2:**
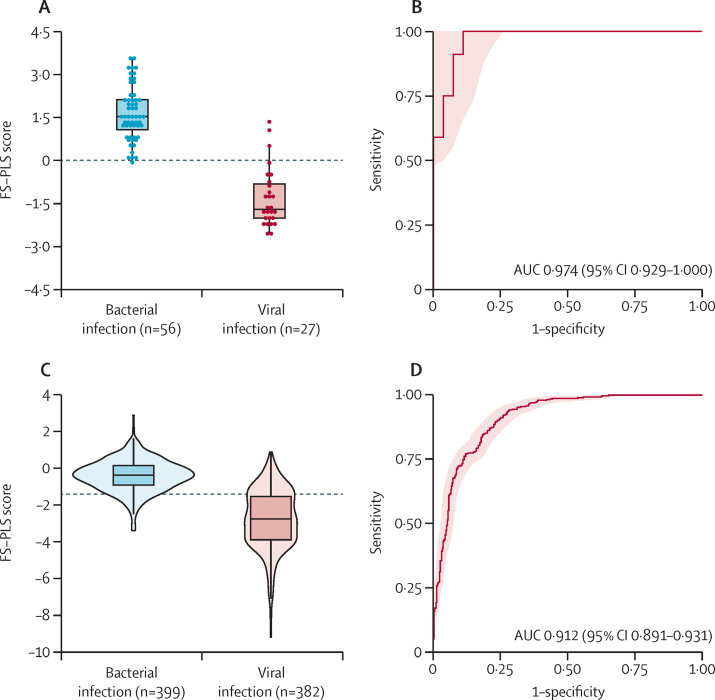
Performance of the FS-PLS signature in the discovery and microarray validation cohorts Boxplot showing the FS-PLS signature score of definite bacterial and viral infections in the discovery cohort (A) and ROC curve of FS-PLS signature for bacterial versus viral infections (B) in the discovery cohort. Boxplot showing the FS-PLS signature score (C) and ROC curve of the FS-PLS signature for bacterial versus viral infections (D) in a previously published microarray cohort. Boxplots show mean and IQR and the dashed line corresponds to the threshold that maximises the Youden's J statistic. In the ROC plots, the shaded areas represent 95% CIs plotted for sensitivity at given in-sample specificities. AUC=area under the curve. FS-PLS=forward selection-partial least squares. ROC=receiver operating characteristic.

The FS-PLS method identified a signature comprising just three genes: *HERC6, IGF1R*, and *NAGK* (appendix p 29). The combination of weighted expression values provided a score for each participant, which yielded an AUROC of 0·974 (95% CI 0·929−1·000), sensitivity of 100% (91·1−100), and specificity of 88·9% (77·8−100) when distinguishing between bacterial and viral infection (figure 2; appendix p 35). *HERC6* and *IGF1R* each had an AUROC of 0·956, whereas *NAGK* had an AUROC of 0·821 (appendix p 30).

The AUROC of 0·974 in discriminating bacterial from viral infection exceeded the performance of either CRP or leukocyte count, which had AUROCs of 0·745 (95% CI 0·622−0·856) and 0·763 (0·647−0·863), respectively (appendix pp 16−17, 30).

To validate the three-gene signature in silico, we tested the signature on adult participants with bacterial (n=399) and viral (n=382) infections, from previously published microarray transcriptomic datasets (appendix pp 31−32).^9^ The FS-PLS model had an AUROC of 0·912 (95% CI 0·891−0·931), sensitivity of 89·5% (76·7−95·5), and specificity of 77·0% (70·7−90·3; figure 2). *HERC6* and *NAGK* each had similar performance, whereas *IGF1R* had reduced performance (appendix p 18). We could not accurately assess the performance of the elastic net signature as only seven of 16 genes were matched to the microarray validation dataset.

To validate the new three-gene signature on a prospective cohort of participants using RT-qPCR, a pre-COVID-19 validation cohort of 228 consecutive BioAID participants presenting to an emergency department over a 6-month period with undifferentiated infection were categorised according to the diagnostic categorisation algorithm (appendix p 12). Of these, 84 had definite or probable bacterial infection, 22 had definite or probable viral infection, 93 had an indeterminate infection, 26 had no infection, and three had an alternate other infection (mycobacterial, fungal, or parasitic; figure 1, appendix p 33). Pathogens identified in the definite infection group in this cohort are recorded in the appendix (p 25). From the 228 participants in the clinical validation cohort, RNA samples for the three-gene RT-qPCR testing were unavailable from four participants, RNA concentrations were too low in 23, and one sample failed the RT-qPCR (figure 1). 200 (88%) of 228 participants in the validation cohort had RT-qPCR data available for analysis. These included 77 participants with definite or probable bacterial infection, and 18 with definite or probable viral infection (appendix p 33).

**Figure 3:**
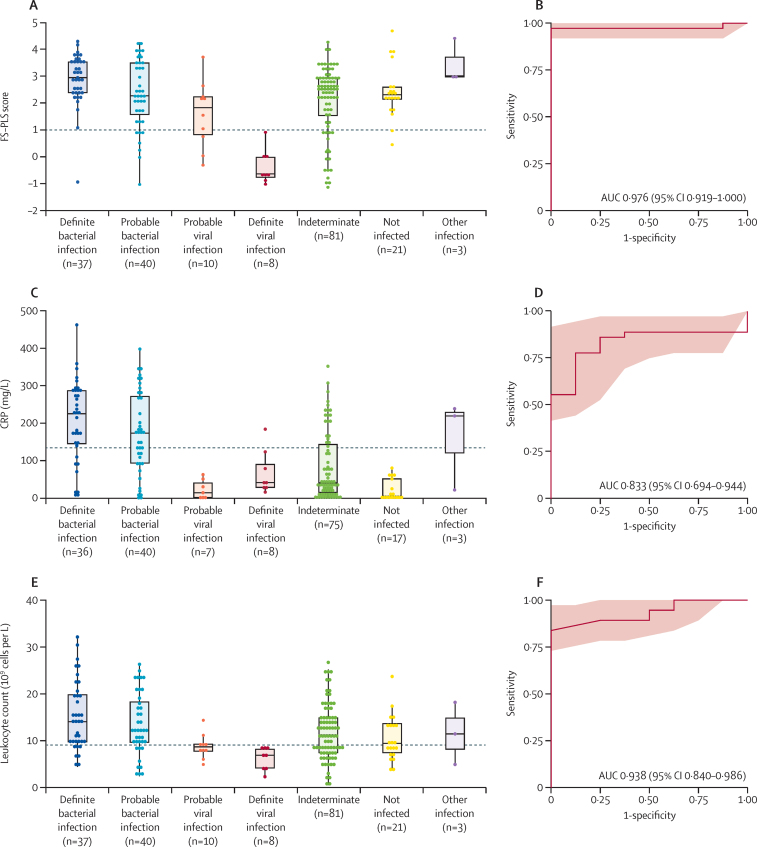
Performance of the FS-PLS signature in the pre-COVID-19 prospective validation cohort Boxplots showing the FS-PLS signature score (A), CRP (C), and leukocyte count (E) in the prospective validation cohort comparing different infection categories. ROC curves of the FS-PLS signature (B) CRP (D), and leukocyte count (F) for definite bacterial versus definite viral comparison. Boxplots show mean and IQR and the horizontal dashed line corresponds to the threshold that maximises the Youden's J statistic. In the ROC plots, the shaded areas represent 95% CIs plotted for sensitivity at given in-sample specificities. AUC=area under the curve. CRP=C-reactive protein. FS-PLS=forward selection-partial least squares. ROC=receiver operating characteristic.

The three-gene signature provided a clear distinction between definite bacterial and viral infections with an AUROC of 0·976 (95% CI 0·919−1·000; figure 3). This discrimination was higher than that for CRP (AUROC 0·833, 0·694−0·944) and leukocyte count (0·938, 0·840−0·986). The sensitivity and specificity of the signature were 97·3% (85·8−99·9) and 100% (63·1−100), respectively, versus 77·8% (60·8−89·9) and 87·5% (47·3−99·7) for CRP, and 83·8% (70·0−93·8) and 100% (63·1−100) for leukocyte count (figure 3; appendix p 19). When combining the definite bacterial and viral groups with those classified as having probable bacterial and viral infections, the signature had an AUROC of 0·841 (0·713−0·934; appendix pp 20−21). For participants with indeterminate infection, as expected, the signature yielded mixed results.

**Figure 4:**
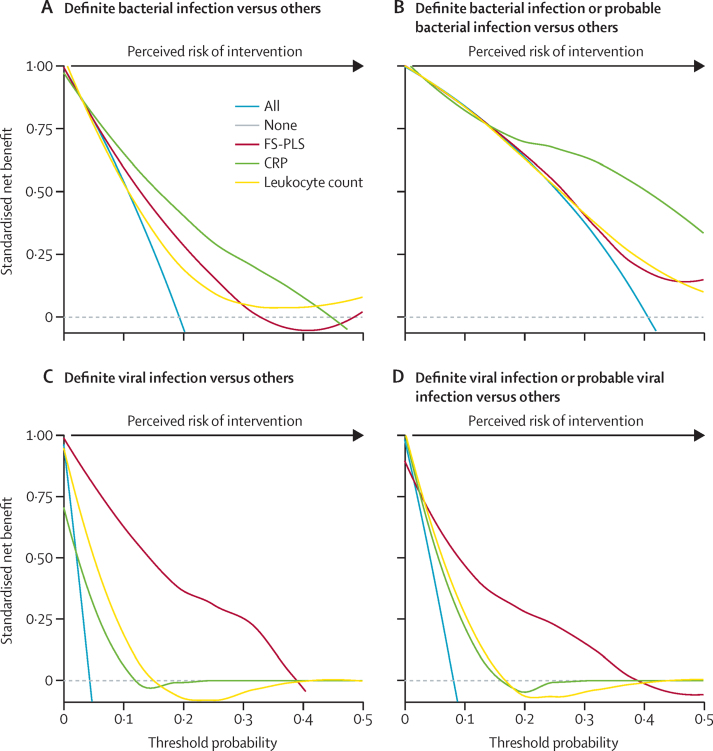
Decision curve analysis in the pre-COVID-19 prospective validation cohort Net benefit for FS-PLS, CRP, or leukocyte count measurements to discriminate between definite bacterial versus others (A), definite and probable bacterial versus others (B), definite viral versus others (C), and definite and probable viral versus others (D). In each analysis, these biomarkers are benchmarked against a treat all or treat none approach. All curves are smoothed using locally estimated scatterplot smoothing. The analysis includes complete cases for which data are available for all three measurements (n=186). CRP=C-reactive protein. FS-PLS=forward selection-partial least squares.

186 participants had data available for decision curve analysis. The new three-gene signature provided the greatest overall net benefit to trigger an intervention for definite or probable viral infection, whereas CRP provided the greatest overall net benefit to trigger an intervention for definite or probable bacterial infection (figure 4).

To test the hypothesis that the three-gene signature can discriminate bacterial infection from COVID-19, we tested blood RNA samples from a further cohort of BioAID participants presenting to the emergency department, comprising 35 participants with bacteraemia recruited before the onset of the first COVID-19 epidemic wave in the UK, and 35 BioAID participants with COVID-19 confirmed by positive SARS-CoV-2 RT-qPCR test (figure 1; appendix p 13). The bacterial group had higher leukocyte and neutrophil counts than the COVID-19 participants but overlapping CRP and lymphocyte counts, and the COVID-19 group was characterised by high mortality (appendix p 34). Pathogens identified in the bacterial group in the COVID-19 validation cohort are recorded in the appendix (p 25). All participants, except one in the COVID-19 validation cohort, had analysable RT-qPCR results.

**Figure 5:**
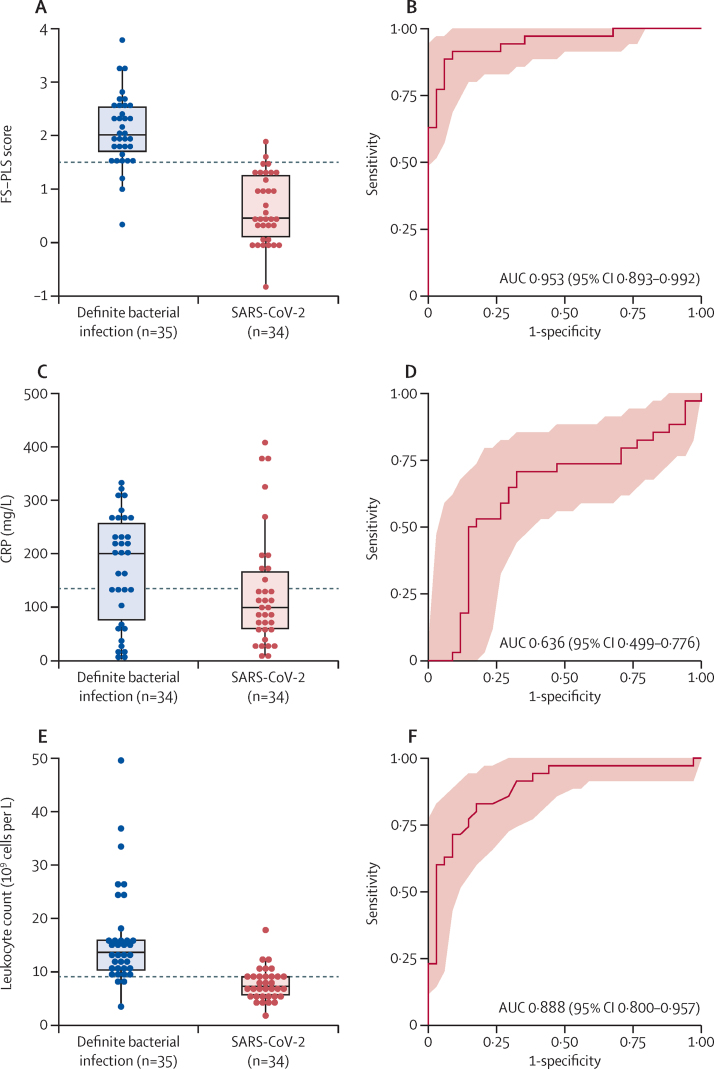
Performance of the FS-PLS signature in the COVID-19 validation cohort Boxplot showing the FS-PLS signature score (A), CRP (C), and leukocyte count (E) in the COVID-19 validation cohort comparing definite bacterial and COVID-19 groups. ROC curve of the FS-PLS signature (B), CRP (D), and leukocyte count (F) for the definite bacterial versus definite COVID-19 comparison. Boxplots show mean and IQR and the horizontal dashed line corresponds to the threshold that maximises the Youden's J statistic. In the ROC plots, the shaded areas represent 95% CIs plotted for sensitivity at given in-sample specificities. AUC=area under the curve. CRP=C-reactive protein. FS-PLS=forward selection-partial least squares. ROC=receiver operating characteristic.

The three-gene signature distinguished the 35 participants with confirmed bacterial infection from 34 participants with confirmed COVID-19 with an AUROC of 0·953 (95% CI 0·893−0·992), sensitivity of 88·6% (73·3−96·8), and specificity of 94·1% (80·3−99·3; figure 5). CRP and leukocyte count achieved an AUROC of 0·636 (0·499−0·776) and 0·888 (0·800−0·957), respectively; sensitivity and specificity were 70·6% (52·5−84·9) and 67·6% (49·5−82·6) for CRP, and 82·9% (66·4−93·4) and 82·4% (65·5−93·2) for leukocyte count. The signature score was not affected by participant age, duration of COVID-19 illness, or COVID-19 severity as assessed by chest radiograph and maximum oxygen requirement (appendix p 22).

## Discussion

There is a pressing need for accessible and rapid diagnostic tools to differentiate between infections in emergency settings. Although much attention has focused on the need to definitively identify bacterial infections and rationalise antibiotic prescribing, the COVID-19 pandemic has highlighted the value of a readily available test that can indicate the strong likelihood of viral infection. Here, we describe a novel three-gene blood transcriptional signature that can discriminate between bacterial and viral infections in adults presenting to an emergency department in the UK. We externally validated this signature against previously published case-control data and a new observational validation cohort, in which the signature discriminated proven bacterial and viral infections with AUROC of 0·976, sensitivity of 97·3%, and specificity of 100%. Notably, our signature also discriminated between bacterial infection and COVID-19 with an AUROC of 0·953, sensitivity of 88·6%, and specificity of 94·1%, whereas CRP levels demonstrated considerable overlap between these groups. Such a test would have been invaluable in the early stages of the COVID-19 pandemic when specific virology tests were not available.

RT-qPCR testing of nasopharyngeal swabs is the current mainstay of COVID-19 diagnosis. Despite widespread use, multiple studies have questioned its reliability as the gold standard owing to low sensitivity when the clinical index of suspicion is high, but also when the time of testing was either too early or too late during the infection course. These doubts led us to explore the use of host gene expression assays.^19^, ^20^ The three-gene signature we discovered could allow for improved stratification of anti-infective prescribing among participants with respiratory illness, contribute to earlier diagnosis, and provide more targeted infection control strategies in the COVID-19 era.

Our study has notable strengths. The three-gene signature was derived using a cohort of adult participants who presented to the emergency department with suspected acute infection, and samples were obtained at the point of presentation, before any treatment or interventions were started. We, therefore, are confident that the signature has been derived at the most relevant timepoint (the point of presentation to secondary care), when a point-of-care test can be of greatest value. By undertaking RNA sequencing on the initial cohort, we were able to exploit the data to design optimised primers for a PCR-based point-of-care test that was validated in two different cohorts, including participants presenting with acute COVID-19 symptoms. The combination of discovery and validation of a signature, including in COVID-19-positive participants, using a tractable PCR-based approach is, to our knowledge, unique. Regarding the genes in the signature, *NAGK* is a salvage enzyme responsible for amino acid metabolism,^21^ *HERC6* belongs to the HERC family of ubiquitin ligases that have been reported to exhibit different antiviral activity when induced by interferon,^22^ and *IGF1R* is an insulin signalling transmembrane tyrosine kinase protein, which is reported to act as an entry receptor for respiratory syncytial virus and also to activate macrophages and redirect phagocytosis.^23^, ^24^ Each gene showed consistent amplification in RT-qPCR, underpinned by RNA sequencing data to inform probe design. Although *HERC6* has been identified as one of a large number of genes that were upregulated in influenza,^25^ to date, the three genes have not been validated nor included in minimal diagnostic signatures.

The prospective validation cohort represented a true-to-life series of consecutive participants presenting to the emergency department with undifferentiated infection, providing insight into how the test might perform in routine practice. All 200 participants in the validation cohort were considered to have infection, had blood cultures taken, and received empiric antibiotics. We used robust clinical and pathological criteria to categorise the 6-month cohort of participants into definite, probable, indeterminate, not infected, and other infection groups to assess the three-gene signature performance in a series of participants with a wide range of diagnoses. We then tested the clinical utility of the signature using decision curve analysis. Consistent with the hypothesis that the three-gene signature primarily reflects the host immune response to viruses, decision curve analysis showed that the signature offered the greatest net benefit (*vs* CRP and leukocyte count) to guide targeting of additional diagnostic tests, antiviral treatment, and infection control measures for proven or probable viral infections, whereas CRP offered the greatest net benefit for targeting of antibacterial treatment for proven or probable bacterial infections. Combining existing and novel biomarkers might, therefore, provide a useful asset in infection management decisions.

There is some evidence to suggest that rapid point-of-care testing has little effect on overall antimicrobial prescribing.^26^, ^27^ However, as the ongoing COVID-19 pandemic shows, the rationale for rapid diagnostic testing goes well beyond antimicrobial stewardship. Indeed, using antimicrobial prescribing as a metric of success undervalues the importance of making a diagnosis and targeting treatments. We believe that a rapid and definitive point-of-care diagnosis of a viral infection could effectively serve as a rule-out for bacterial infection; such a diagnostic test is, therefore, more likely to prevent unnecessary antibiotic prescribing and will target antiviral treatments more effectively, as shown for influenza.^27^

There are some limitations to our study. At the time of recruitment, adults presenting to the emergency department in the UK rarely received a full array of microbiological tests, particularly if viral infection was suspected, contrasting with other settings, such as intensive care. As such, low numbers of confirmed viral infections necessitated a 3-year recruitment period. The signature we discovered differentiated between infections in a UK-based emergency department; we do not know how the test might perform in non-UK settings, or if it could be used in primary care. Whether the test might be of value in co-infections could not be addressed.

Our study has identified various areas for future research. Adult participants with non-infective fever represent a substantial group of emergency department attendees, who might be spared antibiotic exposure if correctly identified, exemplified by the not infected groups in our discovery and validation cohorts. Although promising differences in gene expression were seen, a more comprehensive signature might be required to differentiate this group. Interestingly, a high-performing 16-gene elastic net signature showed great promise at distinguishing between bacterial and viral infections; however, the high number of genes precluded inclusion in a qPCR-based assay. Our future work will address these research questions, and adapt the minimal gene signature for translation to handheld point-of-care testing, where a turn-around time of less than 30 min and low cost (<£5 GBP)^28^, ^29^ might have an even greater effect on infection control, antimicrobial stewardship, and referral to secondary care.

Our work provides proof of principle that small RNA transcript signatures with as few as three gene transcripts can differentiate viral infections, including COVID-19, from other emergency presentations of infection in adults. Whether an enhanced signature to distinguish COVID-19 from other viral and bacterial infections can be derived from new RNA sequencing data is a subject of active research. Rapid point-of-care blood tests might be an asset in future pandemics and assist in the management of participants presenting with undifferentiated infection, particularly as incidence of COVID-19 declines or variants emerge that elude standard tests.^30^ Implications for participant care include improving time to diagnosis, targeted use of proven treatments, appropriate infection control interventions, and consequent decreased length of hospital stay. These represent positive steps for outcome, health-care costs, antimicrobial resistance, and safety within the health-care environment.

## Data sharing

RNA sequencing data are available at https://www.ebi.ac.uk/arrayexpress/ (accession reference E-MTAB-10527). De-identified data that underlie the results in this study will be available on reasonable request to the corresponding author.

## Declaration of interests

We declare no competing interests.

## References

[1] Denny KJ, Gartside JG, Alcorn K, Cross JW, Maloney S, Keijzers G (2019). Appropriateness of antibiotic prescribing in the emergency department. J Antimicrob Chemother.

[2] Mogling R, Meijer A, Berginc N (2020). Delayed laboratory response to COVID-19 caused by molecular diagnostic contamination. Emerg Infect Dis.

[3] Guglielmi G (2020). Fast coronavirus tests: what they can and can't do. Nature.

[4] Gibani MM, Toumazou C, Sohbati M (2020). Assessing a novel, lab-free, point-of-care test for SARS-CoV-2 (CovidNudge): a diagnostic accuracy study. Lancet Microbe.

[5] Chen T, Wu D, Chen H (2020). Clinical characteristics of 113 deceased patients with coronavirus disease 2019: retrospective study. BMJ.

[6] Chen W, Zheng KI, Liu S, Yan Z, Xu C, Qiao Z (2020). Plasma CRP level is positively associated with the severity of COVID-19. Ann Clin Microbiol Antimicrob.

[7] Zeng F, Huang Y, Guo Y (2020). Association of inflammatory markers with the severity of COVID-19: a meta-analysis. Int J Infect Dis.

[8] Ai T, Yang Z, Hou H (2020). Correlation of chest CT and RT-PCR testing for coronavirus disease 2019 (COVID-19) in China: a report of 1014 cases. Radiology.

[9] Sweeney TE, Wong HR, Khatri P (2016). Robust classification of bacterial and viral infections via integrated host gene expression diagnostics. Sci Transl Med.

[10] McHugh L, Seldon TA, Brandon RA (2015). A Molecular host response assay to discriminate between sepsis and infection-negative systemic inflammation in critically ill patients: discovery and validation in independent cohorts. PLoS Med.

[11] Sampson D, Yager TD, Fox B (2020). Blood transcriptomic discrimination of bacterial and viral infections in the emergency department: a multi-cohort observational validation study. BMC Med.

[12] Herberg JA, Kaforou M, Wright VJ (2016). Diagnostic test accuracy of a 2-transcript host RNA signature for discriminating bacterial *vs* viral infection in febrile children. JAMA.

[13] Bhattacharya S, Rosenberg AF, Peterson DR (2017). Transcriptomic biomarkers to discriminate bacterial from nonbacterial infection in adults hospitalized with respiratory illness. Sci Rep.

[14] Tsalik EL, Henao R, Nichols M (2016). Host gene expression classifiers diagnose acute respiratory illness etiology. Sci Transl Med.

[15] Scicluna BP, van Vught LA, Zwinderman AH (2017). Classification of patients with sepsis according to blood genomic endotype: a prospective cohort study. Lancet Respir Med.

[16] Shallcross LJ, Mentzer A, Rahman S, Cooke GS, Sriskandan S, Noursadeghi M (2018). Cohort study protocol: Bioresource in Adult Infectious Diseases (BioAID). Wellcome Open Res.

[17] Coin L (2020). Feature selection–partial least squares code. https://www.github.com/lachlancoin/fspls.

[18] Brown M (2018). Risk model decision analysis. https://mdbrown.github.io/rmda/.

[19] McClain MT, Constantine FJ, Nicholson BP (2021). A blood-based host gene expression assay for early detection of respiratory viral infection: an index-cluster prospective cohort study. Lancet Infect Dis.

[20] Kucirka LM, Lauer SA, Laeyendecker O, Boon D, Lessler J (2020). Variation in false-negative rate of reverse transcriptase polymerase chain reaction-based SARS-CoV-2 tests by time since exposure. Ann Intern Med.

[21] Hinderlich S, Berger M, Schwarzkopf M, Effertz K, Reutter W (2000). Molecular cloning and characterization of murine and human N-acetylglucosamine kinase. Eur J Biochem.

[22] Paparisto E, Woods MW, Coleman MD (2018). Evolution-guided structural and functional analyses of the HERC family reveal an ancient marine origin and determinants of antiviral activity. J Virol.

[23] Griffiths CD, Bilawchuk LM, McDonough JE (2020). IGF1R is an entry receptor for respiratory syncytial virus. Nature.

[24] Han CZ, Juncadella IJ, Kinchen JM (2016). Macrophages redirect phagocytosis by non-professional phagocytes and influence inflammation. Nature.

[25] Andres-Terre M, McGuire HM, Pouliot Y (2015). Integrated, multi-cohort analysis identifies conserved transcriptional signatures across multiple respiratory viruses. Immunity.

[26] Patel SV, Pulcini C, Demirjian A, van Hecke O (2020). Rapid diagnostic tests for common infection syndromes: less haste, more speed. J Antimicrob Chemother.

[27] Brendish NJ, Malachira AK, Armstrong L (2017). Routine molecular point-of-care testing for respiratory viruses in adults presenting to hospital with acute respiratory illness (ResPOC): a pragmatic, open-label, randomised controlled trial. Lancet Respir Med.

[28] Pennisi I, Rodriguez-Manzano J, Moniri A (2021). Translation of a host blood RNA signature distinguishing bacterial from viral infection into a platform suitable for development as a point-of-care test. JAMA Pediatr.

[29] Rodriguez-Manzano J, Malpartida-Cardenas K, Moser N (2021). Handheld point-of-care system for rapid detection of SARS-CoV-2 extracted RNA in under 20 min. ACS Cent Sci.

[30] Food and Drug Administration (2021). Genetic variants of SARS-CoV-2 may lead to false negative results with molecular tests for detection of SARS-CoV-2—letter to clinical laboratory staff and health care providers. https://www.fda.gov/medical-devices/letters-health-care-providers/genetic-variants-sars-cov-2-may-lead-false-negative-results-molecular-tests-detection-sars-cov-2.

