# Supplementary material for: Discovery and validation of a three-gene signature to distinguish COVID-19 and other viral infections in emergency infectious disease presentations: a case-control and observational cohort study
Source: Lancet Microbe. 2021 Nov;2(11):e594–603. doi: 10.1016/S2666-5247(21)00145-2 (PMC8367196; doi:10.1016/S2666-5247(21)00145-2)
Supplement: Supplementary appendix [file mmc1.pdf]

THE LANCET
Microbe

Supplementary appendix

This appendix formed part of the original submission and has been peer reviewed. We post it as supplied by the authors.

Supplement to: Li HK, Kaforou M, Rodriguez-Manzano J, et al. Discovery and validation of a three-gene signature to distinguish COVID-19 and other viral infections in emergency infectious disease presentations: a case-control and observational cohort study. *Lancet Microbe* 2021; published online August 16. https://doi.org/10.1016/S2666-5247(21)00145-2.

SUPPLEMENTARY APPENDIX

to accompany manuscript

Discovery and validation of a three-gene signature to distinguish COVID-19 and other viral infections in emergency infectious disease presentations; a case-control and observational cohort study

Ho Kwong Li MRCP[1,2†], Myrsini Kaforou PhD[1†], Jesus Rodriguez-Manzano PhD[1,3†], Samuel Channon-Wells MMath[1], Ahmad Moniri MEng[4], Dominic Habgood-Coote MSc[1], Rishi K. Gupta MRCP[5], Ewurabena A. Mills BSc[1], Dominique Arancon BSN[6], Jessica Lin MRes[1], Yueh-Ho Chiu PhD[1], Ivana Pennisi MSc[1], Luca Miglietta MSc[1,4], Ravi Mehta MRCP[1], Nelofar Obaray PhD[1], Jethro A. Herberg PhD[1], Victoria J. Wright PhD[1], Pantelis Georgiou PhD[4,7], Laura J. Shallcross PhD[8], Alexander J. Mentzer DPhil[9], Michael Levin PhD[1], Graham S. Cooke DPhil[1], Mahdad Noursadeghi PhD[10] and Shiranee Sriskandan PhD[1,2,3*]

Table of Contents

SUPPLEMENTARY METHODS *4*

Discovery RNA-Seq methods 4

3-gene signature derivation 5

Microarray in silico validation cohort 7

Pre-COVID-19 prospective validation cohort and 3-gene signature evaluation 7

High-throughput qPCR assay for the 3-gene signature in the Pre-COVID-19 prospective validation cohort 8

Decision Curve Analysis of 3-gene signature and other biomarkers in the Pre-COVID-19 prospective validation cohort 9

SUPPLEMENTARY FIGURES *11*

Figure S1. Categorisation algorithm for the Discovery cohort. 11

Figure S2. Categorisation algorithm for the Pre-COVID-19 validation cohort. 12

Figure S3. Categorisation algorithm for the COVID-19 validation cohort. 13

Figure S4. Volcano plot of differentially expressed genes. 14

Figure S5. Elastic net signature performance in the Discovery cohort. 15

Figure S6. Performance of selected FS-PLS genes, compared with CRP and WCC in the Discovery cohort. 16

Figure S7. Performance of selected FS-PLS genes in the Microarray validation dataset 18

Figure S8. Predictive value of the FS-PLS signature score, compared with CRP and WCC in the Pre-COVID-19 validation cohort. 19

Figure S9. Performance of the FS-PLS signature score, compared with CRP and WCC in the Pre-COVID-19 validation cohort, combining definite & probable infection categories.................... 20

Figure S10. Predictive performance of the FS-PLS score assessed against age, duration of illness and severity in the COVID-19 validation cohort... 22

Figure S11. Correction for sequencing plate effect. 23

SUPPLEMENTARY TABLES..24

Table S1. Demographic and clinical data for the Discovery case-control cohort used in RNA-Seq analysis.. 24

Table S2. Pathogens identified in the definite bacterial and definite viral groups for the Discovery, the Pre-COVID-19 validation, and the COVID-19 validation cohorts........................ 25

Table S3. Differential gene expression: comparison of infection versus no infection in the Discovery dataset. 26

Table S4. Genes in the elastic net signature to distinguish bacterial from viral infections........ 28

Table S5. Genes selected by FS-PLS and calculation of score.. 29

Table S6. Performance of the elastic net, FS-PLS genes, individual genes, CRP and WCC prediction in the Discovery cohort. 30

Table S7. Datasets used in the Microarray validation cohort. 31

Table S8. Performance of individual genes and combined FS-PLS score to distinguish bacterial infections from viral infections in the Microarray validation dataset...................................... 32

Table S9. Demographic and clinical data for the Pre-COVID-19 prospective validation cohort. . 33

Table S10. Demographic and clinical data for the COVID-19 case-control validation cohort...... 34

Table S11. Assessment of diagnostic test accuracy by two-way contingency tables................. 35

REFERENCES FOR SUPPLEMENTARY APPENDIX 36

SUPPLEMENTARY METHODS

Discovery RNA-Seq methods

RNA isolation and quantification Whole blood was collected at the time of recruitment in Tempus Blood RNA tubes and total RNA was isolated with the Tempus™ Spin RNA Isolation Kit (ThermoFisher Scientific) according to the manufacturer's instructions. RNA samples were stored at −80 °C until further analysis. After additional DNAse treatment, library preparation and sequencing of 30 million 100bp, paired end reads were conducted using the Illumina's TruSeq® RNA Sample Preparation Kit; ribosomal and globin RNA depletion was performed using the Illumina Ribo-Zero Gold kit and HiSeq 4000 at The Wellcome Centre for Human Genetics in Oxford UK. RNA samples for RT-qPCR were quantified using Qubit™ 4 Fluorometer (ThermoFisher Scientific) and NanoDrop® ND-1000 spectrophotometer (ThermoFisher Scientific) to ensure the integrity and quality of the RNA (260:280 ratio between 1.5 and 1.8).

RNA-Seq quality control and analysis Sample size calculations were performed in R (version 3.4.1) using RNASeqPower (version 1.12.0) (1) based on the following assumptions: (i) RNA-sequencing depth of at least 30million of reads, (ii) a coefficient of variation of 0.3 within the group (iii) probability of 0.05 for a type I error, (iv) probability of 0.1 for a type II error (i.e., a power of 90%) and (v) an effect size of 1.5, indicated a need to include at least 25 (24.7) per group to demonstrate difference in RNA expression. By exceeding this number, we allowed for losses in the experimental pipeline.

The RNA-Seq analysis pipeline consisted of: quality control using FastQC, MultiQC and annotations modified with BEDTools, alignment and read counting using STAR, SAMtools, FeatureCounts and version 89 Ensembl GCh38 genome and annotation.(2-8) Expression levels were normalised and transformed to logarithmic scale normalised using the variance-

stabilising transformation function from the DESeq2 package (9, 10) in R. Genes with minimal variation (less than 10% non-zero values) and genes that had zero reads in more than 90% of the samples were removed. All analyses were undertaken using R version 4.0.0 (R Foundation for Statistical Computing).(11) RNA-Seq data has been uploaded to EMBL-EBI (https://www.ebi.ac.uk/arrayexpress/ - dataset identifier awaited).

Principal Component Analysis (PCA) was implemented as part of our quality control workflow to look for evidence of batch-effect and explore variation of gene expression. This demonstrated substantial batch effect due to sequencing plates used. To correct for this, we used samples from the not-infected group and ComBat (12), an empirical Bayes batch adjustment approach to batch correction, implemented from the Bioconductor package sva in R (13). Inspection of heatmaps (not shown) and principal components (Supplementary Figure S11, p23) demonstrated resolution of the batch effect.

3-gene signature derivation

Raw RNA-Seq data underwent quality control, alignment, gene counting and normalisation. To derive diagnostic gene signatures, we first identified genes that were differentially expressed between bacterial and viral groups using DESeq2. The differential expression model was conditional on age, gender and sequencing plate. Variables with only small expression differences between disease categories are likely to have poor distinguishing power when translated to more diagnostically useful technologies, such as qPCR. To ensure we deselected these variables the differential expression model incorporated a null hypothesis that non-significant genes had $|\log_2 \text{fold-change}| < 0.1$. Adjusted p-values were calculated to optimise test power using data-driven hypothesis weights independent of the p-values (14), implemented with Bioconductor package IHW in R. The genes that were significantly differentially expressed with an absolute $\log_2$ fold-change >1, adjusted p-value <0.05 and sufficient mean expression across the cohort (Supplementary Figure S4, p14) using a model conditional on age,

gender and sequencing plate were subjected to further analysis. The $\log_2$ fold change cut-off was decided to ensure cross-platform reproducibility using PCR, while the adjusted p-value <0.05 cut-off was employed as a less stringent option to allow more genes to enter the feature selection step. To generate lists of differentially expressed genes between the 'no infection' and other groups, a less restrictive null hypothesis was used, with $|\log_2$ fold-change$| = 0$.

Two feature selection methods with different qualities were used to determine small gene sets with the potential for a clinically useful diagnostic tool. The glmnet package (15) was used to implement penalised logistic regression via the elastic net (16). Model parameter alpha was set at 0.95, to mimic the sparse variable selection of lasso whilst allowing grouping of correlated variables. Ten-fold cross-validation was used to optimise remaining parameters. An additional algorithm, Forward Selection - Partial Least Squares (FS-PLS)(17, 18) method was employed to identify a second minimal diagnostic signature, resulting in a logistic regression model. FS-PLS selects for minimally correlated genes and has previously been described in detail (17, 18). The Forward Selection – Partial Least Squares (FS-PLS) method was used to discover gene expression signatures by following an iterative process. The first iteration of the algorithm considers the expression levels of all transcripts (N) and initially fits N univariate regression models. The variable with the highest Maximum Likelihood Estimation and smallest t-test p-value to assess the goodness of fit is selected first (SV1). The algorithm then projects the variation explained by SV1 using Singular Value Decomposition and selects new variables based on the residual (unexplained) variation, until the MLE p-value exceeds a pre-defined threshold. The FS-PLS score was calculated into a single metric using weighted sums of $\log_2$ gene counts from the RNA-Seq analysis. For the validation of the pre-COVID-19 and COVID-19 cohorts, the FS-PLS score was calculated as the weighted sum of RT-qPCR cycle threshold (Ct) values for each gene. To ensure consistent interpretation of the score's direction, the weights were inverted when applied to the RT-qPCR Ct values (Supplementary Table S5, p25).

To evaluate the in-sample model performance we calculated summary statistics of Area Under ROC Curve (AUC), sensitivities and specificities for the elastic net predictions and FS-PLS predictions, when comparing Viral cases ("Controls") with Bacterial cases ("Cases"). This was implemented in the R package pROC (19). The Youden's J statistic (20) was used to determine the most optimal thresholds for sensitivity and specificity. AUC 95% confidence intervals (CI) were calculated using the stratified bootstrap resampling method, with 5,000 resamples and 95% CI for sensitivity and specificity using the exact binomial method. Where confidence intervals were used for ROC curves these were calculated for sensitivities at the given true in-sample specificities. Accuracies of the FS-PLS scores (index test) against definite bacterial or viral categorisation (reference standard) in each cohort (Discovery, Microarray, Pre-Covid-19 & COVID-19) are shown by 2x2 contingency tables (Supplementary Table S11, p35).

Microarray in silico validation cohort

After filtering out patients <18 years of age, and samples from PBMCs, the microarray dataset contained COCONUT co-normalised transcript levels for 399 adult bacterial cases and 382 adult viral cases (Supplementary Table S7, p31). All three genes in the FS-PLS signature were successfully matched. Out-of-sample performance metrics for individual genes are reported (Supplementary Table S8, p32).

Pre-COVID-19 prospective validation cohort and 3-gene signature evaluation

To differentiate viral from bacterial infection with 80% power ($p < 0.05$), using the predicted effect size for all of the genes in the signature (based on RNA-Seq), a group size of 22 was needed. Hence, a pragmatic decision was taken to include an entire unselected six month cohort.

High-throughput qPCR assay for the 3-gene signature in the Pre-COVID-19 prospective validation cohort

TaqMan® Gene Expression assays were purchased from ThermoFisher Scientific targeting HERC6 (assay ID: Hs01111944_m1), NAGK (assay ID: Hs00895035_m1), and IFG1R (assay ID: Hs00609583_m1). Analytical sensitivity and specificity of the three assays were evaluated using transcribed synthetic RNA from gBlocks gene fragments (Integrated DNA Technologies) and human genomic DNA (Promega Corporation), respectively.

We assessed the performance of the signature using a Biomark HD high-throughput qPCR platform and 192.24 Dynamic Array™ integrated fluidic circuit (IFC) following manufacturer instructions. The experimental workflow consisted of 4 steps: reverse transcription, pre-amplification, on-chip gene expression, and data analysis.

Reverse transcription The first step consisted of performing reverse transcription (RT) using the Fluidigm Master Mix, which contains all components required for cDNA synthesis, including buffer dNTPs, ribonuclease inhibitor and an engineered RNA reverse transcriptase (Fluidigm product numbers: #100-5580 and #100-5581). Each RT reaction was performed in 5 µL of final reaction volume with 1 µL of Reverse Transcription Master Mix, 3 µL of RNA-free water and 1 µL of RNA (2.5 pg/µL - 250 ng/µL). The reaction was incubated for 5 minutes at 25°C, 30 minutes at 42°C and 5 minutes at 85°C. This was performed in a conventional real-time qPCR instrument (Roche LightCycler96).

Pre-amplification In a microcentrifuge tube, we combined an equal volume of each of the three 20X TaqMan® Gene Expression assays. The pool was diluted in a buffer, containing 10 mM Tris, pH 8.0, 0.1 mM EDTA, to reach a final concentration of 0.2X. The Pre-Amp reaction was performed in a total volume of 5 µL, which includes 1 µL of Pre-Amp master mix (Fluidigm), 1.25 µL of pooled TaqMan assay mix (0.2X), 1.5 µL of nuclease-free water and 1.25 µL of cDNA for each sample. Pre-Amp cDNA was subjected to a pre-incubation step of 95°C for 2

minutes, followed by 20 cycles of 95°C for 15 seconds and 60°C for 4 minutes. This was performed in a conventional real-time qPCR instrument (Roche LightCycler96). To avoid amplification of genomic DNA, assays were selected with forward and reverse primers separated by a large intron region.

On-chip gene expression The chip required a dilution of each individual Gene Expression assay from 20X (initial concentration) to 10X (final concentration), using a 2X Assay Loading Reagent (Fluidigm PN 100-7611) as dilution component. Each sample was added to a pre-mix solution as follows: 2 µL of TaqMan Universal qPCR Master Mix (2X) (Life Technologies), 0.2 µL of 20X GE Sample Loading Reagent (Fluidigm) and 1.8 µL of cDNA pre-amplified in a final volume of 4 µL. All the reagents, samples and assays were added to the chip before placing it into the RX controller to prime and load. The 192.24 IFC was then placed in the Biomark HD using the GE 192×24 Standard v1.pcl cycling program (Fluidigm) according to the recommended settings given in the manufacturer's 192.24 Fast/Standard Gene Expression Workflow protocol. Each experimental condition was conducted in quadruplicates.

RT-qPCR data analysis The real-time data from RT-qPCR was pre-processed by performing baseline correction and CT values were extracted using a global threshold, as determined by Fluidigm's Real-Time qPCR Software (version 4.1.2). Subsequently, technical replicates were averaged to yield three CT values (one for each gene) per sample. Performance of the assay, to differentiate viral from bacterial and other infection syndromes was evaluated by Area Under ROC Curve (AUC) analyses.

Decision Curve Analysis of 3-gene signature and other biomarkers in the Pre-COVID-19 prospective validation cohort

For each DCA, a logistic regression model was first fitted with the diagnostic test (WCC, CRP or RNA signature) as the only predictor to give the positive predictive value of a test across a range of test thresholds. Net benefit was then calculated as sensitivity × prevalence – (1 –

specificity) × (1 − prevalence) × w where w is the odds at the threshold probability and prevalence is the proportion of patients who met the stated outcome.(21) The threshold probability represents the outcome probability above which a given intervention treatment would be recommended and reflects the risk:benefit ratio for any given intervention or ‘treatment’. Each DCA was benchmarked against the net-benefit derived from a treat all or treat none approach.

SUPPLEMENTARY FIGURES

Figure S1. Categorisation algorithm for the Discovery cohort.

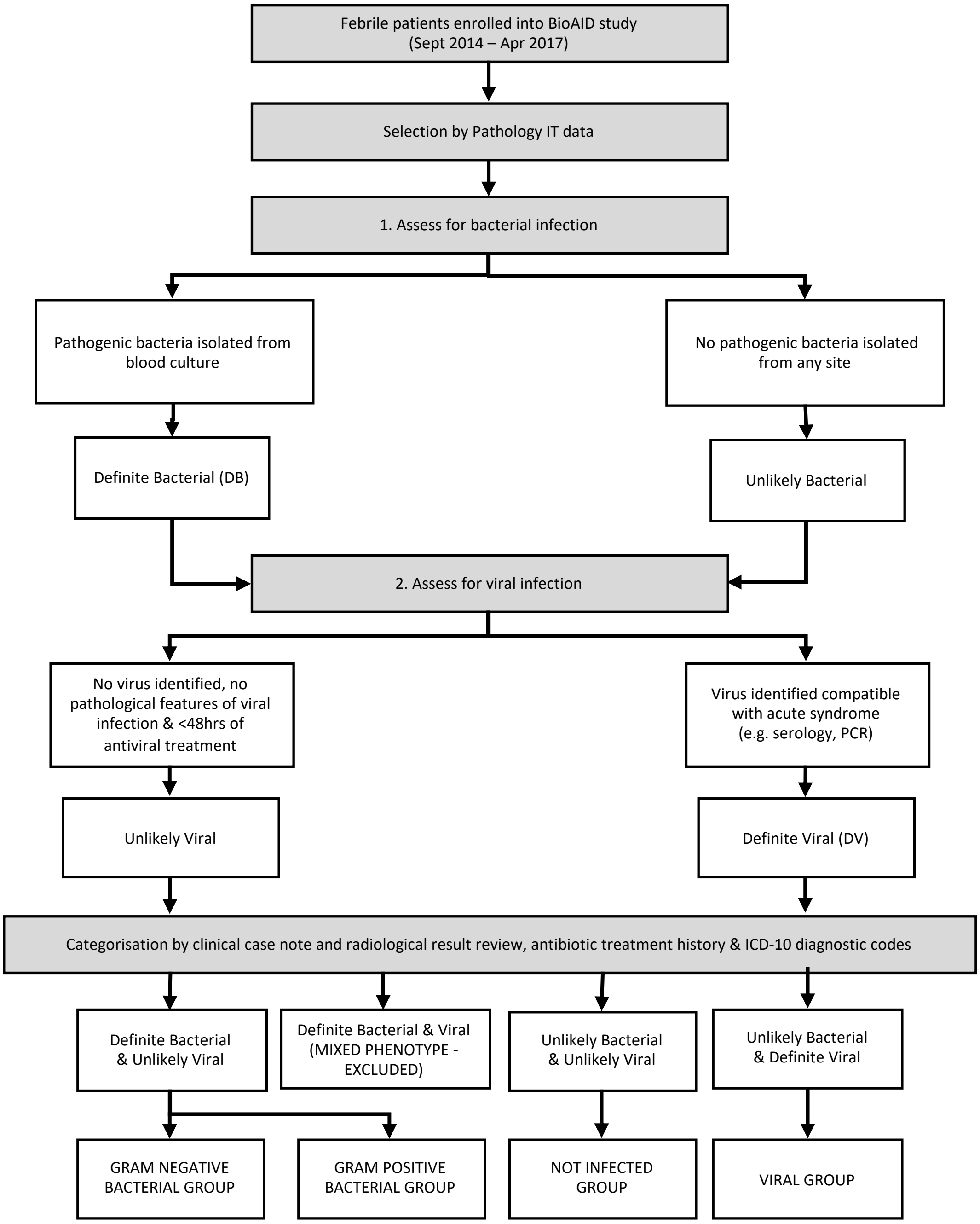

Algorithm used to categorise cases for the discovery cohort into bacterial (comprising both Gram positive and Gram negative), viral and not infected groups. Non-acute or mixed phenotypes were excluded from RNA-Seq analysis.

Figure S2. Categorisation algorithm for the Pre-COVID-19 validation cohort.

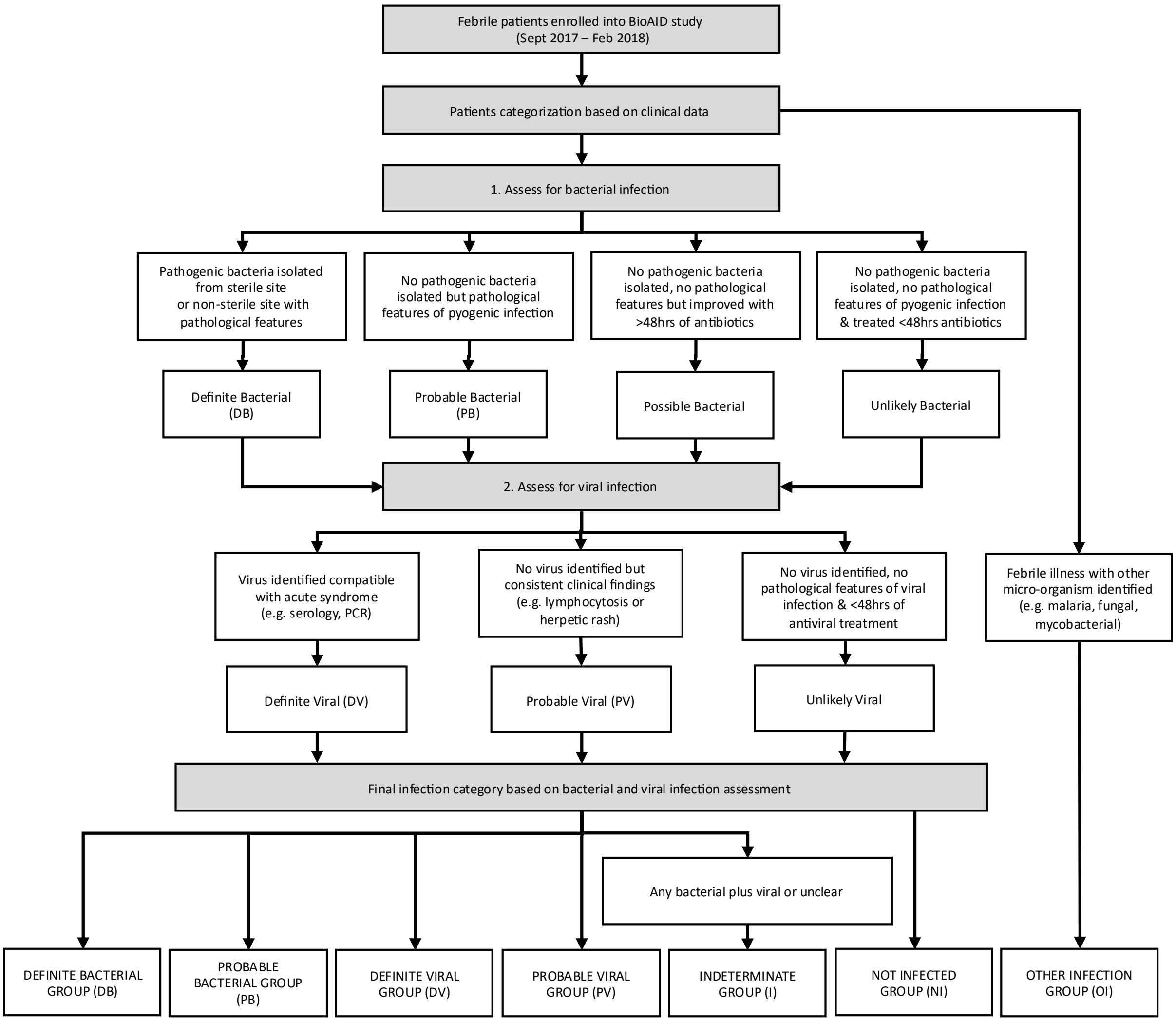

Algorithm used to categorise cases for the pre-COVID-19 validation cohort into definite bacterial, probable bacterial, definite viral, probable viral and indeterminate (co-infection or lacking diagnostic certainty) groups. Non-infected cases and other infections (mycobacterial, fungal or parasitic pathogen cases) were separately categorised.

Figure S3. Categorisation algorithm for the COVID-19 validation cohort.

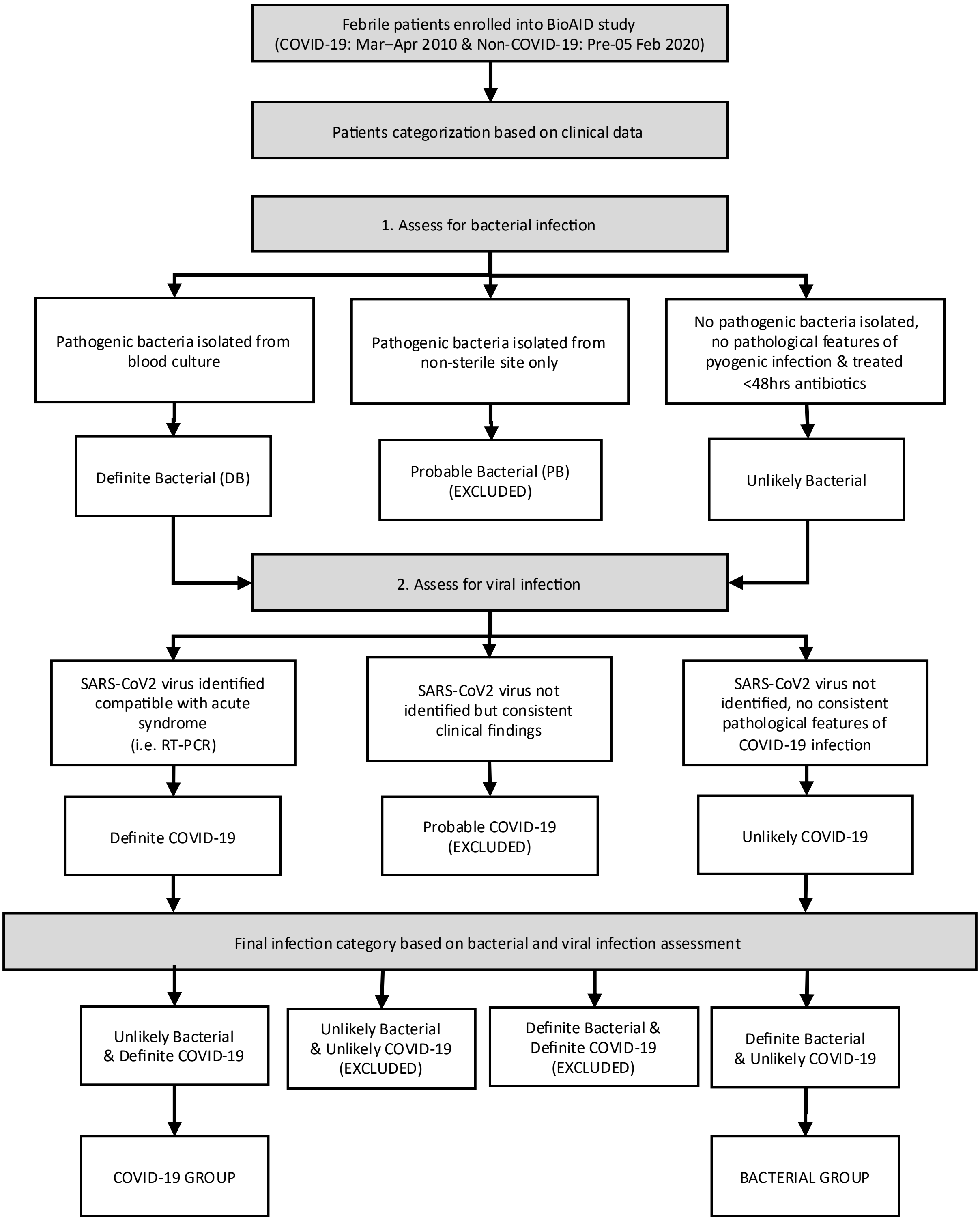

Algorithm used to categorise the COVID-19 validation cohort into bacterial and COVID-19 groups. Probable cases of infection whether bacterial or COVID-19 or where no infection or mixed infections were found, were excluded from analysis.

Figure S4. Volcano plot of differentially expressed genes.

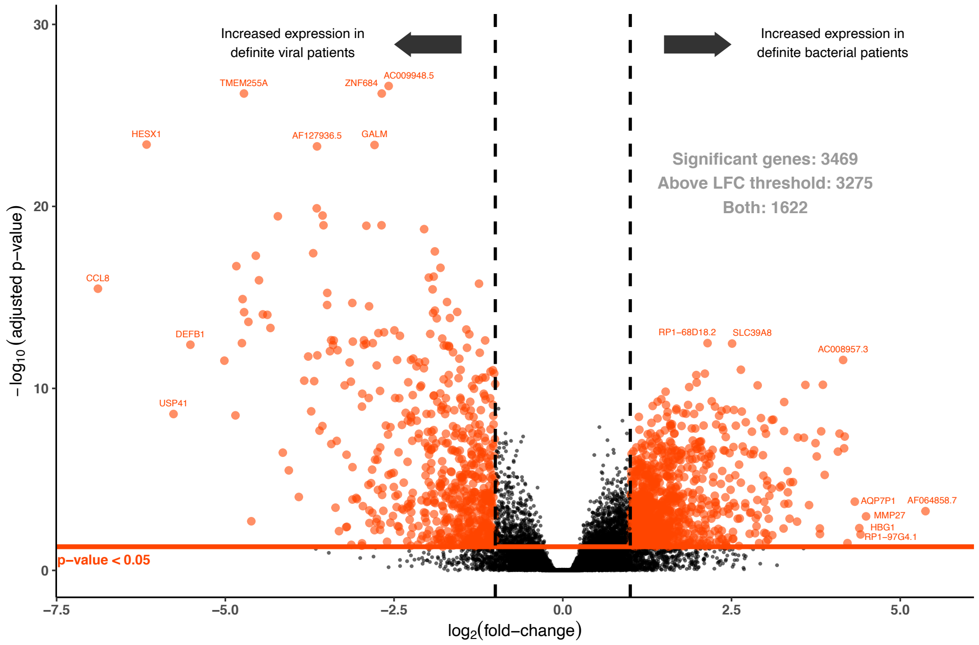

Volcano plot highlighting the significantly differentially expressed genes between patients with bacterial and viral infection. Genes that pass the adjusted p-value threshold of 0.05 and the absolute $\log_2$ fold-change threshold of 1, have been highlighted in red (n=1622).

Figure S5. Elastic net signature performance in the Discovery cohort.

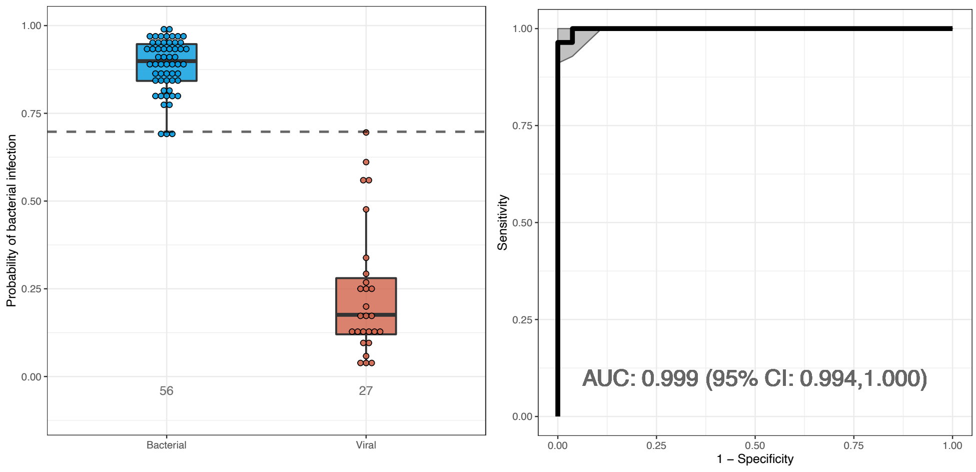

(A) Boxplots of the probability of bacterial over viral infection based on elastic net signature predictions in discovery dataset. Boxplots show mean and interquartile range (IQR), and the horizontal dashed line corresponds to the threshold which maximises the Youden's J statistic. (B) ROC curve of elastic net signature to diagnose bacterial infection (cases) over viral infection (controls) with grey shaded areas representing 95% confidence intervals plotted for sensitivity at given in-sample specificities. Elastic net genes are listed in Supplementary Table S4.

Figure S6. Performance of selected FS-PLS genes, compared with CRP and WCC in the Discovery cohort.

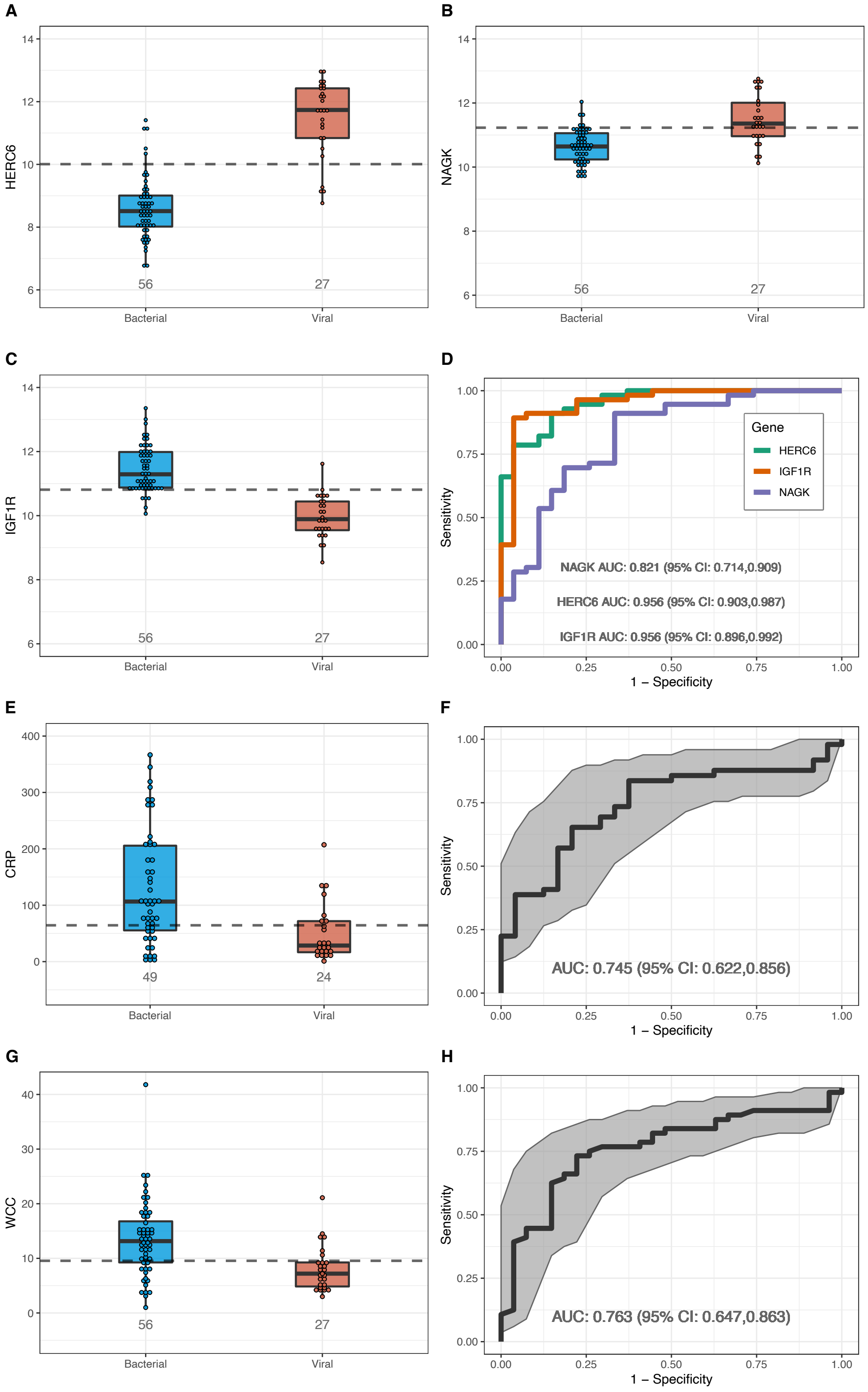

Boxplots showing log_2-scale counts for each of the three genes in the FS-PLS signature: (A) HERC6, (B) NAGK, and (C) IGF1R. ROC curves showing performance of each of the three FS-PLS signature genes in the discovery cohort (D). Performance of CRP demonstrated by (E) boxplots and (F) ROC curve. Performance of leukocyte count (WCC) demonstrated by (G) boxplots and (H) ROC curve. CRP was missing for 10 patients. Boxplots show mean and IQR. The grey shaded areas represent 95% confidence intervals plotted for sensitivity at given in-sample specificities and the horizontal dashed line corresponds to the threshold which maximises the Youden's J statistic. CRP measurements were recorded in mg/L and WCC measurements in 10^9/L.

Figure S7. Performance of selected FS-PLS genes in the Microarray validation dataset.

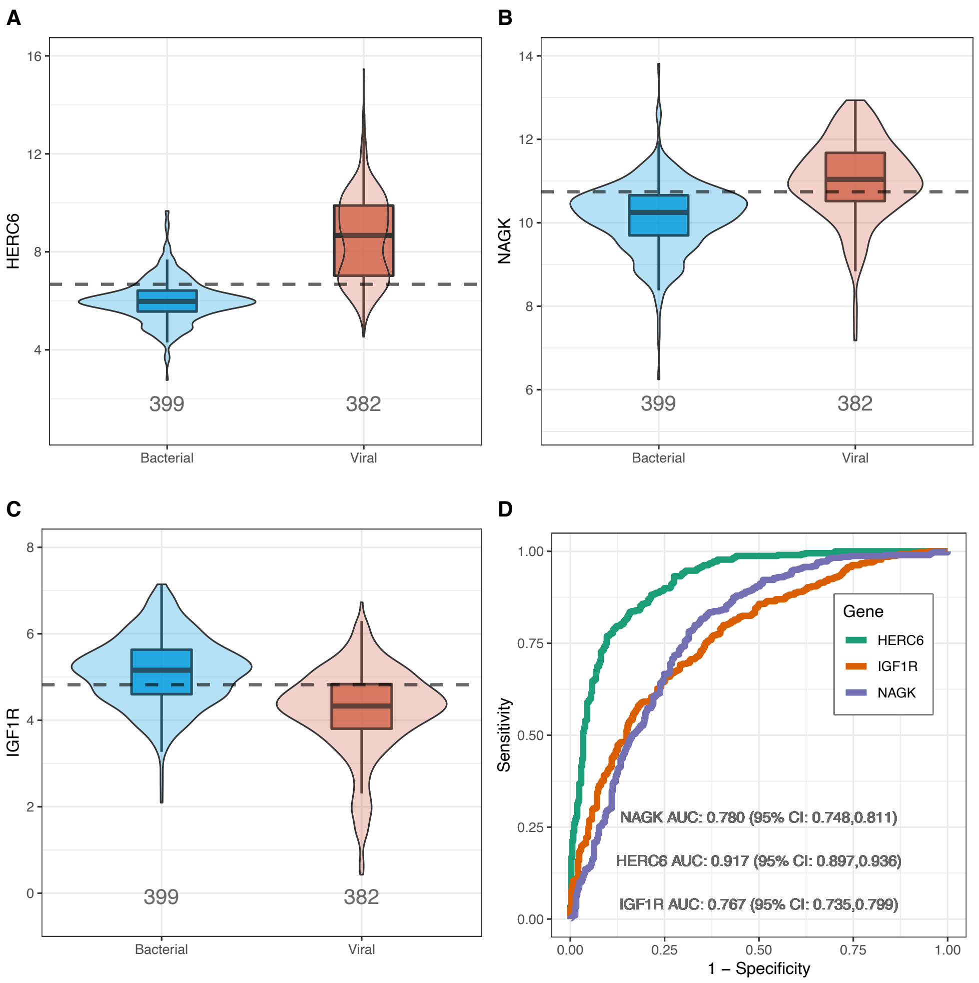

Boxplots of log_2-scale gene expression for each of the selected FS-PLS genes within the microarray validation set: (A) HERC6, (B) NAGK, and (C) IGF1R. (D) ROC curves for each gene with 95% CI. Boxplots show mean and IQR and the horizontal dashed line corresponds to the threshold which maximises the Youden's J statistic.

Figure S8. Predictive value of the FS-PLS signature score, compared with CRP and WCC in the Pre-COVID-19 validation cohort.

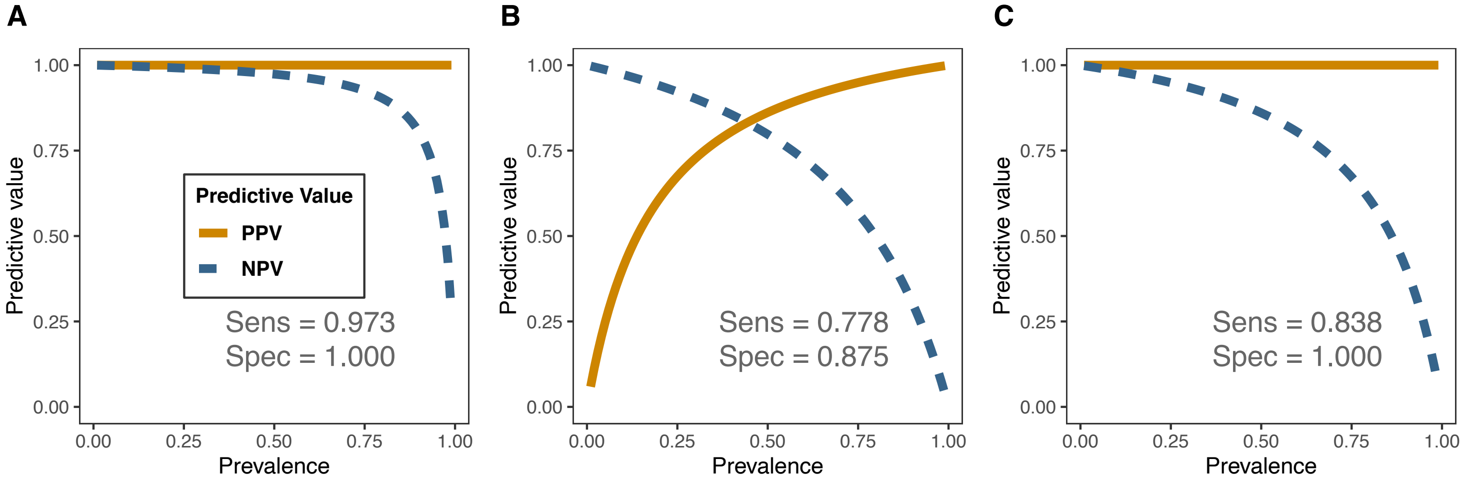

Predictive value of (A) the FS-PLS signature score when differentiating Definite Bacterial from Definite Viral infections compared with (B) the predictive value of CRP and, (C) the predictive value of leucocyte count (WCC). CRP measurements were recorded in mg/L and WCC measurements in 10^9/L.

Figure S9. Performance of the FS-PLS signature score, compared with CRP and WCC in the Pre-COVID-19 validation cohort, combining definite & probable infection categories.

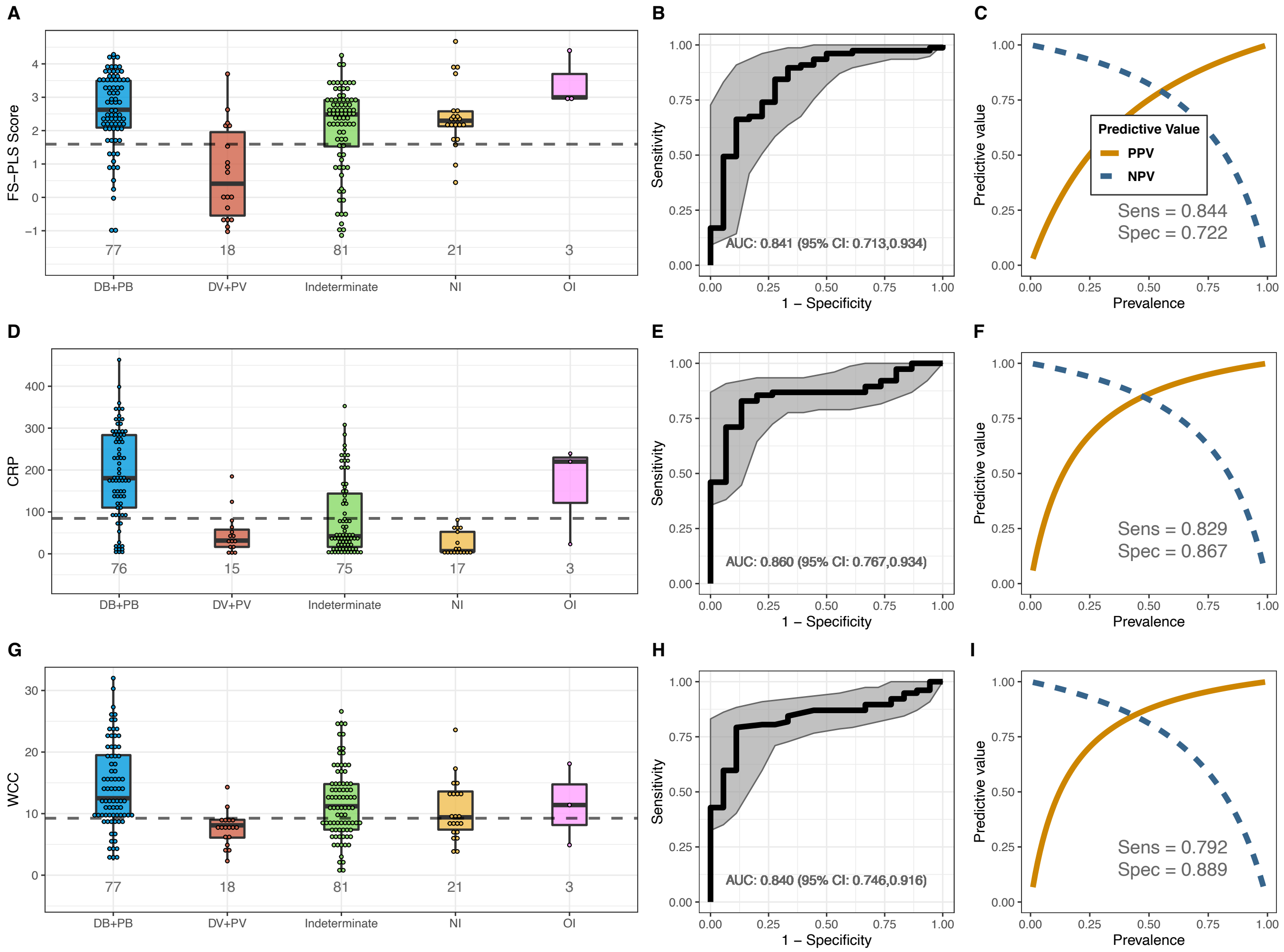

Boxplot (A) showing FS-PLS signature score in the validation cohort when definite and probable cases of bacterial or viral infection are combined. (B) ROC curve showing performance of FS-PLS score to differentiate definite and probable bacterial groups from definite and probable viral groups with 95% CI and (C) predictive value of FS-PLS score. These are compared with (D) boxplots showing CRP, (E) ROC curve of CRP, (F) predictive value of CRP and (G) boxplots showing leukocyte count (WCC), (H) ROC curve of WCC, (I) predictive value of WCC in same patient cohorts. CRP was missing for 14 patients. Boxplots show mean and IQR. The grey shaded areas represent 95% confidence intervals plotted for sensitivity at given in-sample specificities and the horizontal dashed line corresponds to the threshold which maximises the Youden's J statistic. CRP measurements were recorded in mg/L and WCC measurements in 10^9/L.

Figure S10. Predictive performance of the FS-PLS score assessed against age, duration of illness and severity in the COVID-19 validation cohort.

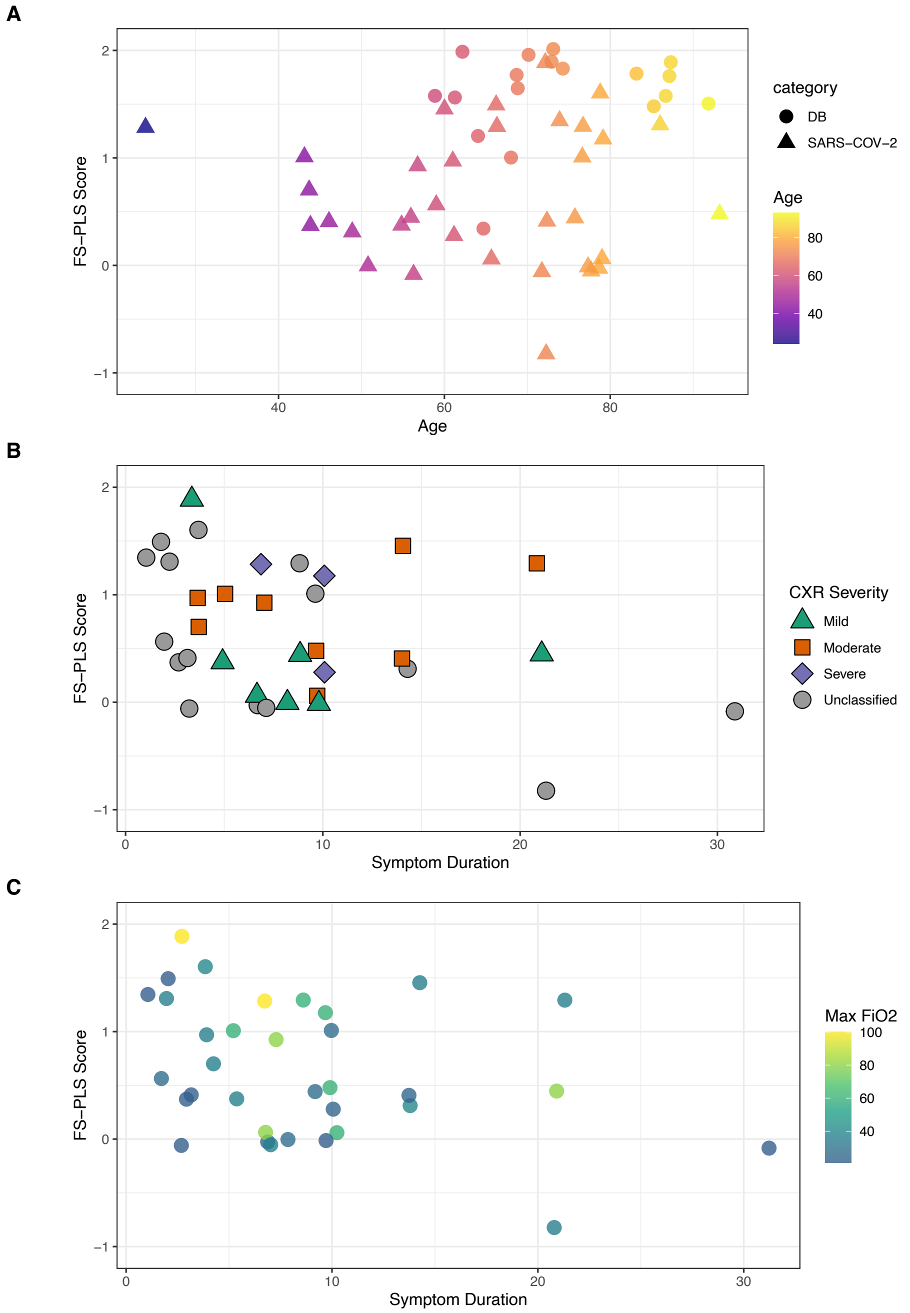

Dot plots showing FS-PLS score assessed against: (A) age, (B) duration of illness with severity as indicated by chest radiograph, and (C) duration of illness with severity as indicated by maximum FiO_2 requirement in first 24h of illness. Panels (B) and (C) include only those with COVID-19.

Figure S11. Correction for sequencing plate effect.

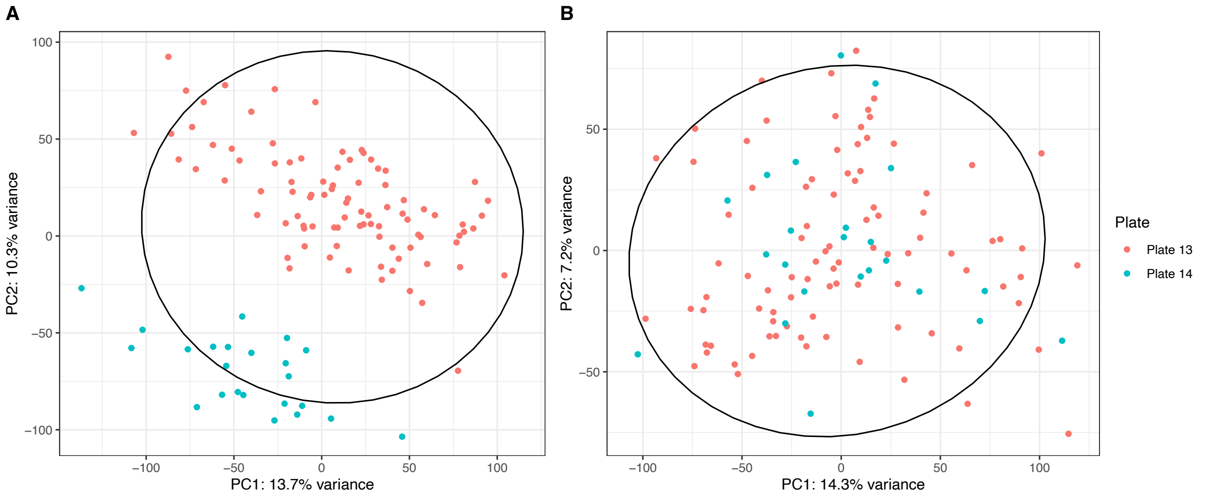

(A) Pre-plate effect correction Principal Component Analysis plot illustrating the separation of samples in the 2nd principal component, depending on sequencing plate. (B) Post plate effect correction PCA plot demonstrating no determinable batch effect in the 1st or 2nd principal components after correction.

SUPPLEMENTARY TABLES

Table S1. Demographic and clinical data for the Discovery case-control cohort used in RNA-Seq analysis.

	DISCOVERY RNA-Seq COHORT		
CHARACTERISTIC	Bacterial Group (N = 56)	Viral Group (N = 27)	Not Infected Group# (N = 27)
Age - years‡			
Median (IQR)	71 (52.3 - 80.8)	44 (26.0 - 54.0)	48 (31.0 - 70.0)
Range	25 - 95	19 - 68	19 - 96
Gender - frequency (%)			
Male sex	24 (42.9)	15 (55.6)	17 (63.0)
Baseline comorbidities - frequency (%)‡			
No	22 (39.3)	21 (77.8)	17 (63.0)
Yes	34 (60.7)	6 (22.2)	10 (37.0)
Admission temperature - Celsius*			
Median (IQR)	38.1 (36.9 - 39.0)	38.4 (37.8 - 38.9)	37.0 (36.5 - 37.3)
Range	35.5 - 40.2	36.9 - 39.8	35.6 - 37.9
Admission laboratory tests - median (IQR)			
Leucocytes‡ (10^9/L)	13.2 (9.2 - 17.3)	7.2 (4.6 - 9.4)	8.9 (6.3 - 12.1)
Neutrophils‡ (10^9/L)	11.3 (7.5 - 14.8)	5.1 (3.6 - 7.6)	6.1 (4.5 - 8.3)
Lymphocytes (10^9/L)	0.9 (0.5 - 1.2)	0.8 (0.5 - 1.2)	1.6 (0.9 - 2.5)
C-reactive protein‡* (mg/L)	106.5 (54.1 - 206.3)	28.5 (16.6 - 72.0)	3.4 (1.3 - 14.3)
Inpatient Mortality - frequency (%)			
Died	9.0 (16.1)	0.0 (0.0)	0.0 (0.0)

Signature discovery was derived from Bacterial and Viral groups only. Two cases in the Not-infected group were separate admissions from one patient

‡ Significant difference between Bacterial and Viral groups where $p<0.001$ using unpaired T-test, or, for Baseline comorbidities, Fischer's exact test (≥1 comorbidity)

* Sixteen temperature readings missing from time of admission (Bacterial group, 9; Viral group, 1; Not-infected group, 6). Thirteen CRP results missing from time of admission (Bacterial group, 7; Viral group, 3; Not-infected group, 3)

Table S2. Pathogens identified in the definite bacterial and definite viral groups for the Discovery, the Pre-COVID-19 validation, and the COVID-19 validation cohorts.

<table>
<tr><th>COHORT</th><th>GROUP</th><th colspan="8">PATHOGEN</th></tr>
<tr><td rowspan="6">DISCOVERY</td><td rowspan="2">Gram Positive</td><td>Staphylococcus aureus</td><td>Streptococcus pneumoniae</td><td>Group C/G Streptococci</td><td>Viridans Streptococci</td><td>Group B Streptococci</td><td>Enterococcus species</td><td>Listeria species</td><td>Lactobacillus paracasei</td></tr>
<tr><td>11</td><td>4</td><td>4</td><td>3</td><td>1</td><td>5</td><td>1</td><td>1</td></tr>
<tr><td rowspan="2">Gram Negative</td><td colspan="3">Escherichia coli</td><td colspan="3">Klebsiella pneumoniae</td><td colspan="2">Enterobacter cloacae</td></tr>
<tr><td colspan="3">28</td><td colspan="3">1</td><td colspan="2">1</td></tr>
<tr><td rowspan="2">Viral</td><td>Influenza A</td><td>Adenovirus</td><td>Measles</td><td>Dengue</td><td>HSV</td><td>EBV</td><td>VZV</td><td>HMPV or rhinovirus or parainfluenza</td></tr>
<tr><td>13</td><td>5</td><td>3</td><td>2</td><td>2</td><td>1</td><td>1</td><td>3</td></tr>
<tr><td rowspan="4">PRE-COVID-19 VALIDATION</td><td rowspan="2">Bacterial</td><td>Escherichia coli</td><td>Streptococcus species</td><td>Klebsiella pneumoniae</td><td>Pseudomonas aeruginosa</td><td>Staphylococcus aureus</td><td>Other Gram Negatives</td><td>Other Gram Positives</td><td>Polymicrobial</td></tr>
<tr><td>16</td><td>7</td><td>4</td><td>2</td><td>3</td><td>3</td><td>2</td><td>1</td></tr>
<tr><td rowspan="2">Viral</td><td colspan="2">Influenza A/B</td><td colspan="2">Acute HIV</td><td colspan="2">VZV</td><td>Measles</td><td>RSV</td></tr>
<tr><td colspan="2">4</td><td colspan="2">1</td><td colspan="2">1</td><td>1</td><td>1</td></tr>
<tr><td rowspan="4">COVID-19 VALIDATION</td><td rowspan="2">COVID-19</td><td colspan="8">SARS-Cov2</td></tr>
<tr><td colspan="8">35</td></tr>
<tr><td rowspan="2">Bacterial</td><td>Escherichia coli</td><td>Staphylococcus aureus</td><td>Viridans Streptococci</td><td>Group C/G Streptococci</td><td>Proteus mirabilis</td><td>Serratia marscesens</td><td>Enterobacter cloacae</td><td>Clostridium perfringens</td></tr>
<tr><td>25</td><td>3</td><td>2</td><td>1</td><td>1</td><td>1</td><td>1</td><td>1</td></tr>
</table>

Table S3. Differential gene expression: comparison of infection versus no infection in the Discovery dataset.

Ensembl Gene ID	Gene Symbol	Base Mean	Log_2 Fold Change	Adjusted p	Description (Source: HGNC)
ENSG00000102794	IRG1	20.78	4.33	1.67E-08	immunoresponsive 1 homolog (mouse)
ENSG00000169245	CXCL10	662.03	4.61	1.67E-08	chemokine (C-X-C motif) ligand 10
ENSG00000152766	ANKRD22	1400.57	3.2	2.18E-08	ankyrin repeat domain 22
ENSG00000078098	FAP	102.69	3.74	3.46E-08	fibroblast activation protein, alpha
ENSG00000226004	RP11-10J5.1	63.35	4.05	6.32E-08	
ENSG00000154099	DNAAF1	21.79	6.7	1.07E-07	dynein, axonemal, assembly factor 1
ENSG00000139572	GPR84	606.05	3.78	1.15E-07	G protein-coupled receptor 84
ENSG00000170439	METTL7B	58.03	3.23	4.10E-07	methyltransferase like 7B
ENSG00000225492	GBP1P1	549.43	3.9	2.89E-06	guanylate binding protein 1, interferon-inducible pseudogene 1
ENSG00000078081	LAMP3	495.96	3.91	4.76E-06	lysosomal-associated membrane protein 3
ENSG00000110079	MS4A4A	671.12	2.4	3.06E-05	membrane-spanning 4-domains, subfamily A, member 4A
ENSG00000121797	CCRL2	320.02	2.27	4.15E-05	chemokine (C-C motif) receptor-like 2
ENSG00000137869	CYP19A1	67.88	3.46	4.15E-05	cytochrome P450, family 19, subfamily A, polypeptide 1
ENSG00000135424	ITGA7	233.57	2.79	7.61E-05	integrin, alpha 7
ENSG00000169248	CXCL11	26.81	3.74	0.000121	chemokine (C-X-C motif) ligand 11
ENSG00000131979	GCH1	2623.53	2.07	0.000124	GTP cyclohydrolase 1
ENSG00000228863	RP11-404F10.2	222.83	3.12	0.000124	
ENSG00000120217	CD274	3302.88	2.59	0.000156	CD274 molecule
ENSG00000155886	SLC24A2	4.99	-4.72	0.000213	solute carrier family 24 ($Na^+/K^+/Ca^{2+}$ exchanger), member 2
ENSG00000249173	LINC01093	115.63	3.1	0.000213	long intergenic non-protein coding RNA 1093
ENSG00000224789	AC012363.4	112.33	2.77	0.00029	
ENSG00000123610	TNFAIP6	3770.83	2.43	0.000374	tumor necrosis factor, alpha-induced protein 6
ENSG00000254420	RP11-452H21.1	87.47	3.08	0.000374	
ENSG00000151364	KCTD14	11.2	3.8	0.000389	potassium channel tetramerization domain containing 14
ENSG00000158488	CD1E	3.99	-2.73	0.000409	T-cell surface glycoprotein CD1e molecule, (membrane-associated)
ENSG00000162772	ATF3	152.29	2.7	0.000433	activating transcription factor 3
ENSG00000255397	AC022182.2	13.51	2.72	0.000433	
ENSG00000259354	RP11-519G16.3	8.02	4.51	0.000433	
ENSG00000079215	SLC1A3	809.74	3.07	0.000442	solute carrier family 1 (glial high affinity glutamate transporter), member3

ENSG00000118113	MMP8	6870.79	3.42	0.000609	matrix metallopeptidase 8 (neutrophil collagenase)
ENSG00000079385	CEACAM1	3520.1	2.4	0.000644	carcinoembryonic antigen-related cell adhesion molecule 1
ENSG00000184979	USP18	470.17	3.53	0.000966	ubiquitin specific peptidase 18
ENSG00000002549	LAP3	4880.33	2.29	0.001349	leucine aminopeptidase 3
ENSG00000108691	CCL2	95.01	4.2	0.001349	chemokine (C-C motif) ligand 2
ENSG00000108700	CCL8	71.58	4.88	0.001477	chemokine (C-C motif) ligand 8
ENSG00000198785	GRIN3A	133.51	2.88	0.001477	glutamate receptor, ionotropic, N-methyl-D-aspartate 3A
ENSG00000163666	HESX1	59.89	3.84	0.00155	HESX homeobox 1
ENSG00000136689	IL1RN	2096.32	2.41	0.00165	interleukin 1 receptor antagonist
ENSG00000255355	AP000640.2	18.09	2.99	0.001753	
ENSG00000105707	HPN	17.55	3.02	0.002347	hepsin
ENSG00000196141	SPATS2L	749.75	2.72	0.002347	spermatogenesis associated, serine-rich 2-like
ENSG00000117228	GBP1	13600.44	2.4	0.002706	guanylate binding protein 1, interferon-inducible
ENSG00000105967	TFEC	4518.85	2.04	0.002888	transcription factor EC
ENSG00000163746	PLSCR2	70.71	2.06	0.003186	phospholipid scramblase 2
ENSG00000186583	SPATC1	11.99	3.98	0.003225	spermatogenesis and centriole associated 1
ENSG00000258227	CLEC5A	839.02	2.14	0.003342	C-type lectin domain family 5, member A
ENSG00000279400	AC008957.3	143.3	3.17	0.003646	
ENSG00000163959	SLC51A	80.58	3.1	0.003782	solute carrier family 51, alpha subunit
ENSG00000145365	TIFA	2140.38	2.04	0.00388	TRAF-interacting protein with forkhead-associated domain
ENSG00000183578	TNFAIP8L3	29.43	3.13	0.00388	tumor necrosis factor, alpha-induced protein 8-like 3
ENSG00000188313	PLSCR1	8485.55	1.83	0.003935	phospholipid scramblase 1
ENSG00000133101	CCNA1	83.42	2.73	0.004398	cyclin A1
ENSG00000131203	IDO1	740.15	3.08	0.00467	indoleamine 2,3-dioxygenase 1
ENSG00000255221	CARD17	683.07	2.42	0.005602	caspase recruitment domain family, member 17
ENSG00000272567	RP11-73K9.3	35.92	2.1	0.007699	
ENSG00000265531	FCGR1C	483.06	2.26	0.007735	Putative high affinity immunoglobulin gamma Fc receptor IC
ENSG00000115267	IFIH1	4805.03	2.16	0.007828	interferon induced with helicase C domain 1
ENSG00000254649	RP11-452H21.2	10.52	3.31	0.008662	
ENSG00000163958	ZDHHC19	77.03	3.13	0.009897	zinc finger, DHHC-type containing 19

Log_2 fold increase in Infection versus No Infection is indicated by pink shading. Log_2 fold decrease in Infection versus No Infection is indicated by blue shading. Intensity of shading indicates increasing fold difference.

Table S4. Genes in the elastic net signature to distinguish bacterial from viral infections.

Gene Symbol	Description (Source: HGNC)	Elastic Net Coefficient	Base Mean	Log_2 Fold Change	Log_2 Fold Change Standard Error	Adjusted p
BAK1	BCL2 antagonist/killer 1	0.18	347	-1.03	0.17	5.80E-06
BCAT1	branched chain amino acid transaminase 1	-0.06	1070	1.01	0.27	7.00E-03
CMC2	C-X9-C motif containing 2	0.03	236	-1.03	0.14	0.00E+00
ZNF684	zinc finger protein 684	0.71	207	-2.68	0.22	0.00E+00
TRPM6	transient receptor potential cation channel subfamily M member 6	-0.01	1205	1.69	0.23	0.00E+00
AGO2	argonaute RISC catalytic component 2	-0.04	3390	1.09	0.17	2.00E-07
MICALCL	MICAL C-terminal like	-0.01	339	1.21	0.29	2.00E-03
SLC39A8	solute carrier family 39 member 8	-0.08	1512	2.51	0.3	0.00E+00
IGF1R	insulin like growth factor 1 receptor	-0.41	2507	1.4	0.19	0.00E+00
NEXN	nexilin F-actin binding protein	0.02	444	-1.76	0.22	0.00E+00
DDIT3	DNA damage inducible transcript 3	0.01	387	-1.01	0.15	1.00E-07
CMTM4	CKLF like MARVEL transmembrane domain containing 4	-0.17	210	1.4	0.2	0.00E+00
ISG15	ISG15 ubiquitin like modifier	0	1563	-4.33	0.51	0.00E+00
ORM1	orosomucoid 1	-0.07	166	3.59	0.48	0.00E+00
AL109809.1	pseudogene similar to F830045P16Rik	-0.31	138	1.37	0.2	0.00E+00
CKAP2LP1	CKAP2L pseudogene 1	-0.09	113	1.59	0.23	0.00E+00

Table S5. Genes selected by FS-PLS and calculation of score.

		Gene Expression Differences[α]				Calculation of FS-PLS scores
**Gene Symbol**	**Description (Source: HGNC)**	**Base Mean**	**Log_2 Fold Change**	**Log_2 Fold Change Standard Error**	**Adjusted p**	**FS-PLS weights[β]**
NAGK	N-acetylglucosamine kinase	2123	-1.01	0.16	5.00E-07	0.076193749
HERC6	HECT & RLD domain containing E3 ubiquitin protein ligase family member 6	1337	-2.91	0.29	0.00E+00	-0.734076039
IGF1R	insulin like growth factor 1 receptor	2507	1.4	0.19	0.00E+00	0.629199527

[α] Gene expression differences compare definite bacterial group with definite viral

[β] FS-PLS score calculated by multiplying weights by gene counts (log_2) for RNA-Seq data. For RT-qPCR, the FS-PLS score was calculated by multiplying the inverted weights by the RT-qPCR cycle threshold (Ct) values for each gene.

Table S6. Performance of the elastic net, FS-PLS genes, individual genes, CRP and WCC prediction in the Discovery cohort.

	**Elastic Net**	**FS-PLS**	**NAGK**	**HERC6**	**IGF1R**	**CRP**	**WCC**
**Area under ROC curve**	0.999	0.974	0.821	0.956	0.956	0.745	0.763
**AUC 95% CI**	(0.994-1.000)	(0.929-1.000)	(0.714-0.909)	(0.903-0.987)	(0.896-0.992)	(0.622-0.856)	(0.647-0.863)
**Sensitivity (%)**	96.4	100	91.1	91.1	89.3	83.7	73.2
**Sensitivity 95% CI**	(92.9-100)	(91.1-100)	(62.5-98.2)	(73.2-100)	(82.1-98.2)	(40.8 - 91.8)	(48.2 - 87.5)
**Specificity (%)**	100	88.9	66.7	85.2	96.3	62.5	77.8
**Specificity 95% CI**	(96.3-100)	(77.8-100)	(51.9-92.6)	(77.8 - 100)	(85.2-100)	(54.2 - 100)	(63 - 96.3)

Table S7. Datasets used in the Microarray validation cohort.

Accession	Author	Tissue	Platforms	Demographic	Bacteria	Viruses	Number healthy	Number bacterial	Number viral
**GSE20346**	Parnell	Whole blood	GPL6947	Adults with CAP	Unknown bacterial pneumonia	Influenza	36	12	8
**GSE40012**	Parnell	Whole blood	GPL6947	Adults with CAP	Unknown bacterial pneumonia	Influenza	18	36	11
**GSE60244**	Suarez	Whole blood	GPL10558	Adults hospitalized with LRTI	Gram-positive and atypical	Influenza, RSV, MPV	40	22	71
**GSE63990**	Tsalik	Whole blood	GPL571	Adults with ARI	Multiple	Multiple	0	70	115
**E-MEXP-3589**	Almansa	Whole blood	GPL10332	Adults with COPD with infection	Gram-positive, Gram-negative, atypical	Influenza, RSV, MPV	4	4	5
**GSE13015**	Pankla	Whole blood	GPL6106, GPL6947	Adults with bacterial sepsis	Burkholderia pseudomallei and others	-	20	60	0
**GSE28750**	Sutherland	Whole blood	GPL570	Adults with comm-acquired bacterial sepsis	Multiple bacteria	-	20	10	0
**GSE29161**	Thuny	Whole blood	GPL6480	Adults with native valve-infected endocarditis	Staphylococcus and Streptococcus	-	5	5	0
**GSE33341**	Ahn	Whole blood	GPl571	Adults with septic bloodstream infections	S. aureus or E. coli	-	43	51	0
**GSE40586**	Lill	Whole blood	GPL6244	Bacterial meningitis	Multiple bacteria	-	18	15	0
**GSE42834**	Bloom	Whole blood	GPL10558	Bacterial pneumonia	Unknown	-	118	19	0
**GSE57065**	Cazalis	Whole blood	GPL570	Adults with bacterial septic shock	Multiple bacteria	-	25	82	0
**GSE69528**	Conejero	Whole blood	GPL10558	Adults with bacterial sepsis	Burkholderia pseudomallei and others	-	55	83	0
**E-MTAB-3162**	van de Weg	Whole blood	GPL570	Indonesian patients >14 yrs old with severe dengue	-	Dengue	15	0	30
**GSE17156**	Zaas	Whole blood	GPL571	Volunteers with viral challenge peak symptoms	-	Influenza, RSV, rhinovirus	56	0	27
**GSE21802**	Bermejo-Martin	Whole blood	GPL6102	Adults with septic influenza	-	Influenza (H1N1)	4	0	12
**GSE27131**	Berdal	Whole blood	GPL6244	Adults with septic influenza with mechanical ventilation	-	Influenza (H1N1)	7	0	7
**GSE68310**	Zhai	Whole blood	GPL10558	Adults with ARIs	-	Influenza & rhinovirus	243	0	211

(Ref 22)

Table S8. Performance of individual genes and combined FS-PLS score to distinguish bacterial infections from viral infections in the Microarray validation dataset.

	**HERC6**	**IGF1R**	**NAGK**	**FS-PLS**
**Bacterial Cases (N)**	399	399	399	399
**Viral Cases (N)**	382	382	382	382
**Area Under ROC curve**	0.917	0.767	0.780	0.912
**AUC 95% CI**	(0.897 - 0.936)	(0.735 - 0.799)	(0.748 - 0.811)	(0.891 - 0.931)
**Sensitivity (%)**	83	67.2	81.2	89.5
**Sensitivity 95% CI**	(75.7 - 94.2)	(55.6 - 82.7)	(74.7 - 87.7)	(76.7 - 95.5)
**Specificity (%)**	86.4	75.4	66.8	77.0
**Specificity 95% CI**	(73 - 92.4)	(58.1 - 85.6)	(58.4 - 73.3)	(70.7 - 90.3)

Table S9. Demographic and clinical data for the Pre-COVID-19 prospective validation cohort.

	PRE-COVID-19 PROSPECTIVE VALIDATION COHORT (N=200 with analysable RT-qPCR data)						
CHARACTERISTIC	Definite Bacterial Group (N = 37)	Probable Bacterial Group (N = 40)	Probable Viral Group (N = 10)	Definite Viral Group (N = 8)	Indeterminate Group (N = 81)	Not Infected Group (N = 21)	Other Infection Group (N = 3)
Age - years							
Median (IQR)	65 (53.5 - 79.0)	63.5 (43.0 - 75.0)	41.5 (26.0 - 59.3)	55.5 (32.0 - 66.3)	66 (46.0 - 75.5)	58 (43.5 - 69.5)	74 (-)
Range	20 - 91	21 - 91	25 - 85	23 - 78	18 - 91	21 - 94	70 - 84
Gender - frequency (%)							
Male sex	14 (37.8)	25 (62.5)	6 (60.0)	5 (62.5)	42 (51.9)	9 (42.9)	2 (66.7)
Baseline comorbidities - frequency (%)							
No	13 (35.1)	16 (40.0)	9 (90.0)	2 (25.0)	27 (33.3)	12 (57.1)	2 (66.7)
Yes	24 (64.9)	24 (60.0)	1 (10.0)	6 (75.0)	54 (66.7)	9 (42.9)	1 (33.3)
Admission temperature - Celsius*							
Median (IQR)	37.9 (36.9 - 39.0)	37.4 (36.4 - 37.9)	38.1 (36.6 - 38.3)	38.2 (37.0 - 38.8)	37.5 (36.6 - 38.2)	36.5 (35.6 - 37.0)	38.1 (-)
Range	32.0 - 39.4	31.3 - 39.9	34.8 - 38.9	36.5 - 39.1	31.5 - 39.5	33.3 - 38.0	37.9 - 38.2
Admission laboratory tests - median (IQR)							
Leucocytes (10^9/L)	14.0 (9.7 - 20.1)	12.3 (9.4 - 18.7)	8.7 (7.2 - 9.7)	6.9 (3.9 - 8.2)	11.2 (7.4 - 14.9)	9.4 (7.0 - 13.7)	11.4 (-)
Neutrophils (10^9/L)	11.1 (7.7 - 17.3)	9.5 (6.9 - 14.3)	6.3 (5.4 - 8.1)	5.0 (2.6 - 6.6)	9.1 (5.3 - 12.6)	7.4 (4.3 - 10.4)	10.5 (-)
Lymphocytes (10^9/L)	0.9 (0.6 - 1.5)	1.1 (0.5 - 1.7)	1.1 (0.6 - 1.5)	0.9 (0.6 - 1.1)	1.1 (0.7 - 1.6)	1.8 (0.7 - 2.4)	0.6 (-)
C-reactive protein* (mg/L)	226 (142.4 - 289)	174 (92.3 - 279)	15.7 (1.2 - 52.0)	42.6 (29 - 113)	41.9 (14.8 - 149)	6.4 (4.9 - 56.0)	131.3 (-)
Inpatient Mortality - frequency (%)							
Died	3 (8.1)	2 (5.0)	0 (0.0)	0 (0.0)	2 (2.5)	2 (9.5)	0 (0.0)

* Four temperature readings were missing from time of admission (Indeterminate group, 1; Not-infected group, 2; Other infection group, 1). Thirteen CRP results missing from the first 24 hours of admission (Definite Bacterial, 1; Probable Viral, 3; Indeterminate group, 4; Not infected group 4; Other Infection group 1)

Table S10. Demographic and clinical data for the COVID-19 case-control validation cohort.

	COVID-19 VALIDATION COHORT	
CHARACTERISTIC	Bacterial Group (N = 35)	COVID-19 Group (N = 34)
Age - years		
Median (IQR)	73.0 (64.0 - 85.0)	66.0 (55.8 - 77.0)
Range	26 - 92	24 - 93
Gender - frequency (%)		
Male sex	20 (57.1)	25 (73.5)
Baseline comorbidities - frequency (%)		
No	16 (45.7)	4 (11.8)
Yes	19 (54.3)	30 (88.2)
Admission temperature - Celsius*		
Median (IQR)	38.1 (37.1 - 38.9)	38.0 (36.9 - 38.3)
Range	34.5 - 40.3	33.9 - 40.4
Admission laboratory tests - median (IQR)		
Leucocytes‡ (10^9/L)	13.6 (10.2 - 15.9)	7.2 (5.6 - 9.1)
Neutrophils‡ (10^9/L)	11.7 (9.0 - 14.6)	5.9 (4.2 - 7.8)
Lymphocytes (10^9/L)	0.7 (0.4 - 1.2)	0.7 (0.6 - 1.1)
C-reactive protein* (mg/L)	200.0 (66.9 - 264.0)	99.4 (58.7 - 172.0)
Inpatient Mortality - frequency (%)		
Died	6 (17.1)	14 (41.2)

‡ Significant difference between bacterial and viral groups where $p<0.001$ using unpaired T-test

* One temperature reading was missing from time of admission (1 in bacterial group). One CRP result was missing from time of admission (1 in bacterial group)

Table S11. Assessment of diagnostic test accuracy by two-way contingency tables.

DISCOVERY COHORT		Category	
		**Bacterial**	**Viral**
**FS-PLS Score**	**Above threshold**	56	3
	**Below threshold**	0	24

MICROARRAY VALIDATION COHORT		Category	
		**Bacterial**	**Viral**
**FS-PLS Score**	**Above threshold**	357	88
	**Below threshold**	42	294

PRE-COVID-19 VALIDATION COHORT		Category	
		**DB**	**DV**
**FS-PLS Score**	**Above threshold**	36	0
	**Below threshold**	1	8

COVID-19 VALIDATION COHORT		Category	
		**DB**	**SARS-CoV-2**
**FS-PLS Score**	**Above threshold**	31	2
	**Below threshold**	4	32

Two by two contingency tables comparing performance of the FS-PLS score against the clinical coding as bacterial or viral infection in the Discovery and Microarray cohorts; Definite Bacterial (DB) or Definite Viral (DV) in the Pre-COVID-19 Validation cohort; and DB or SARS-CoV-2 in the COVID-19 Validation cohort. The Youden's J statistic (20) was used to determine the most optimal thresholds for sensitivity and specificity.

REFERENCES FOR SUPPLEMENTARY APPENDIX

1. Therneau T, Hart S, Kocher J. Calculating samples size estimates for RNA Seq studies. R package version 1.30.0. 2020 [

2. Cock PJ, Fields CJ, Goto N, Heuer ML, Rice PM. The Sanger FASTQ file format for sequences with quality scores, and the Solexa/Illumina FASTQ variants. Nucleic Acids Res. 2010;38(6):1767-71.

3. Ewels P, Magnusson M, Lundin S, Kaller M. MultiQC: summarize analysis results for multiple tools and samples in a single report. Bioinformatics. 2016;32(19):3047-8.

4. Quinlan AR. BEDTools: The Swiss-Army Tool for Genome Feature Analysis. Curr Protoc Bioinformatics. 2014;47:11 2 1-34.

5. Dobin A, Davis CA, Schlesinger F, Drenkow J, Zaleski C, Jha S, et al. STAR: ultrafast universal RNA-seq aligner. Bioinformatics. 2013;29(1):15-21.

6. Li H, Handsaker B, Wysoker A, Fennell T, Ruan J, Homer N, et al. The Sequence Alignment/Map format and SAMtools. Bioinformatics. 2009;25(16):2078-9.

7. Liao Y, Smyth GK, Shi W. featureCounts: an efficient general purpose program for assigning sequence reads to genomic features. Bioinformatics. 2014;30(7):923-30.

8. Yates AD, Achuthan P, Akanni W, Allen J, Allen J, Alvarez-Jarreta J, et al. Ensembl 2020. Nucleic Acids Res. 2020;48(D1):D682-D8.

9. Love MI, Huber W, Anders S. Moderated estimation of fold change and dispersion for RNA-seq data with DESeq2. Genome Biol. 2014;15(12):550.

10. Anders S, Huber W. Differential expression analysis for sequence count data. Genome Biol. 2010;11(10):R106.

11. Team RC. R: A language and environment for statistical computing. Vienna, Austria2020 [Available from: https://www.r-project.org] (Accessed 07 January 2021).

12. Johnson WE, Li C, Rabinovic A. Adjusting batch effects in microarray expression data using empirical Bayes methods. Biostatistics. 2007;8(1):118-27.

13. Leek JT, Johnson WE, Parker HS, Fertig EJ, Jaffe AE, Zhang Y, Storey JD and Torres LC. sva: Surrogate Variable Analysis. R package version 3.36.0. 2020 [Available from: https://bioconductor.org/packages/release/bioc/html/sva.html] (Accessed 07 January 2021).

14. Ignatiadis N, Klaus B, Zaugg JB, Huber W. Data-driven hypothesis weighting increases detection power in genome-scale multiple testing. Nat Methods. 2016;13(7):577-80.

15. Friedman J, Hastie T, Tibshirani R. Regularization Paths for Generalized Linear Models via Coordinate Descent. J Stat Softw. 2010;33(1):1-22.

16. Zou H, Hastie T. Regularization and variable selection via elastic net. J R Statist Soc B. 2005;67:301-20.

17. Coin L. Feature Selection - Partial Least Squares code 2020 [Available from: https://www.github.com/lachlancoin/fspls] (Accessed 07 January 2021).

18. Herberg JA, Kaforou M, Wright VJ, Shailes H, Eleftherohorinou H, Hoggart CJ, et al. Diagnostic Test Accuracy of a 2-Transcript Host RNA Signature for Discriminating Bacterial vs Viral Infection in Febrile Children. JAMA. 2016;316(8):835-45.

19. Robin X, Turck N, Hainard A, Tiberti N, Lisacek F, Sanchez JC, et al. pROC: an open-source package for R and S+ to analyze and compare ROC curves. BMC Bioinformatics. 2011;12:77.

20. Youden WJ. Index for rating diagnostic tests. Cancer. 1950;3(1):32-5.

21. Vickers AJ, van Calster B, Steyerberg EW. A simple, step-by-step guide to interpreting decision curve analysis. Diagn Progn Res. 2019;3:18.

22. Sweeney TE, Wong HR, Khatri P. Robust classification of bacterial and viral infections via integrated host gene expression diagnostics. Sci Transl Med. 2016;8(346):346ra91.
